# Proposing Effective Ecotoxicity Test Species for Chemical Safety Assessment in East Asia: A Review

**DOI:** 10.3390/toxics12010030

**Published:** 2023-12-30

**Authors:** Jin Wuk Lee, Ilseob Shim, Kyunghwa Park

**Affiliations:** Research of Environmental Health, National Institute of Environmental Research, Incheon 404-708, Republic of Korea; similseob@korea.kr (I.S.); mirrorpark@korea.kr (K.P.)

**Keywords:** test species, environmental risk assessment, ecotoxicity, biomarker, alternative approach

## Abstract

East Asia leads the global chemical industry, but environmental chemical risk in these countries is an emerging concern. Despite this, only a few native species that are representative of East Asian environments are listed as test species in international guidelines compared with those native to Europe and America. This review suggests that *Zacco platypus*, *Misgurnus anguillicaudatus*, *Hydrilla verticillata*, *Neocaridina denticulata* spp., and *Scenedesmus obliquus*, all resident to East Asia, are promising test species for ecotoxicity tests. The utility of these five species in environmental risk assessment (ERA) varies depending on their individual traits and the state of ecotoxicity research, indicating a need for different applications of each species according to ERA objectives. Furthermore, the traits of these five species can complement each other when assessing chemical effects under diverse exposure scenarios, suggesting they can form a versatile battery for ERA. This review also analyzes recent trends in ecotoxicity studies and proposes emerging research issues, such as the application of alternative test methods, comparative studies using model species, the identification of specific markers for test species, and performance of toxicity tests under environmentally relevant conditions. The information provided on the utility of the five species and alternative issues in toxicity tests could assist in selecting test species suited to study objectives for more effective ERA.

## 1. Introduction

**Figure 1:**
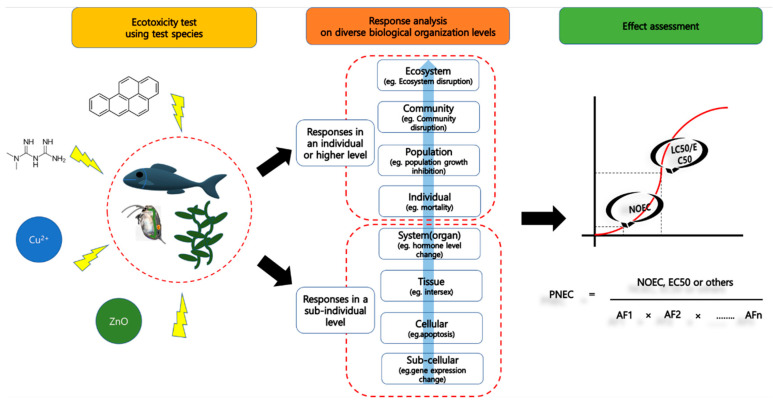
Conceptual diagram of chemical effect assessment and ecotoxicity test species role in the assessment.

Choosing suitable ecotoxicity test species is a critical initial step in effect assessment for environmental risk assessment (ERA), as responses at different levels of biological organization to chemical exposure can serve as predictive evidence of chemical impacts [1]. Sub-individual level responses, known as biomarkers, are useful in effect assessment as they can function as early warning signals for adverse outcomes [2]. Additionally, responses at the organism level or higher, such as mortality, behavior changes, and a decrease in population, can provide direct evidence for assessing chemical effects on the environment [1,2] (Figure 1). Specifically, the responses of test species native to the environment under study can be crucial evidence in ERA. This is because the physiological traits of these species adapted to the region can be helpful for the prediction of the chemical effects on that region, especially if site-specific information is required, and if a few key species determine the function and structure of the aquatic ecosystem [3].

Nevertheless, challenges exist in applying these species to assessment of chemical effects. One issue is interspecies variability in chemical toxicity. Research has demonstrated that each species in an ecosystem may respond differently to chemical exposure [4]. While uncertainty factors and species sensitivity distributions have been applied to ERA to address this, the lack of toxicity mechanisms that explain interspecies differences introduces uncertainty [1,4]. Thus, identifying test species with diverse traits in response to chemical exposure is crucial. Moreover, as an ecosystem comprises various species interconnected by food chains, it would be more effective to select multiple species from different trophic levels rather than relying on a single species. In summary, to perform effective ERA for chemicals, there is a need to develop diverse ecotoxicity test species, taking into account interspecific differences and roles within food chains.

East Asia is a significant player in global industrial chemical production. In particular, South Korea, Japan, and China, situated in East Asia, lead the world’s industrial chemical sales markets. From 2011 to 2021, China, Japan, and South Korea ranked first, fourth, and fifth in chemical sales, collectively accounting for 51% of global market sales in 2021 [5]. As the chemical market expands in these countries, the emphasis on chemical safety management is increasing. Consequently, the governments of South Korea, Japan, and China have enacted chemical regulatory acts/laws, including South Korea’s Act on Registration and Evaluation of Chemicals and Consumer Chemical Products and Biocides Safety Control Act, Japan’s Chemical Substance Control Law, and China’s Ministry of Ecology and Environment Order No. 12 [6]. Based on the principle of “no data, no market,” these acts require all manufacturers or importers of chemicals to register or obtain approval for their products from regulatory authorities, providing proof of safety to humans and the environment through human risk assessment and ERA. For ERA, regulatory authorities require manufacturers/importers to submit ecotoxicity test data conducted following international standard test guidelines (e.g., OECD test guidelines), which recommend several model/test species for ecotoxicity testing.

In these guidelines, most ecotoxicity study protocols have been conducted using only a single or a few model species. Typically, toxicity tests for ERA recommend model species such as *Danio rerio*, *Daphnia magna*, *Pseudokirchneriella subcapitata*, among others [7,8]. The rationale for using these model species is that they are cost-effective as they have been cultured under laboratory conditions for several years. Furthermore, the test methods for these species are typically better established compared with those for non-model species, and they are more easily integrated into toxicity evaluations [3]. However, most of these model species inhabit temperate climates and originate from North America, South Asia, and Europe. For East Asia, there are only a few recommended model species (e.g., *Oryzias latipes*). Therefore, there is a need for increased efforts to identify resident test species in East Asia that would be useful for ERA.

In this context, this study aims to propose promising test species, each possessing a variety of traits useful for ERA in East Asia, and to analyze the utility of these species for ERA. Additionally, by examining trends in ecotoxicity tests focusing on alternative approaches in ERA, this study identifies and suggests challenges and future research directions for enhancing the utility of test species in ERA. This research will be beneficial for identifying and applying reliable test species for effect assessment in ERA.

## 2. Method

### 2.1. Literature Searching Strategy

To find literature for promising test species in chemical ecotoxicity test, scientific manuscripts, reports, web-sites, books were searched in Scopus (https://www.scopus.com (accessed on 1 January 2021–31 December 2023)), Google Scholar (https://scholar.google.com (accessed on 1 January 2021–31 December 2023)), Pubmed (https://pubmed.ncbi.nlm.nih.gov (accessed on 1 January 2021–31 December 2023)), NDSL (http://www.ndsl.or.kr (accessed on 1 January 2021–31 December 2023)). Searching keywords involve test species, environmental risk assessment, ecotoxicity, biomarker, alternative toxicity test. Adverse effects such as endocrine disruption, liver toxicity, developmental toxicity, neurotoxicity, reproduction toxicity, histological malformation, mortality, metabolism disruption, oxidative stress, immune toxicity, and so forth were us ed as key words. The chemical keywords include metal (e.g., copper), biocides (e.g., benzisothiazolinone), pharmaceuticals (e.g., metformin), surfactants (e.g., sodium dodecyl sulfate), nanoparticles (e.g., carbon nanotube), hydrocarbons (e.g., benzo(*a*)pyrene), perfluoroalkyl acids (e.g., PFOS), and others. Test species names such as *Scenedesmus obliquus* and others were used as keywords to cite references. By extension, the reference list of cited manuscripts was analyzed and re-cited, expanding searching sources.

### 2.2. Selection Criteria for Freshwater Test Species of East Asia

**Figure 2:**
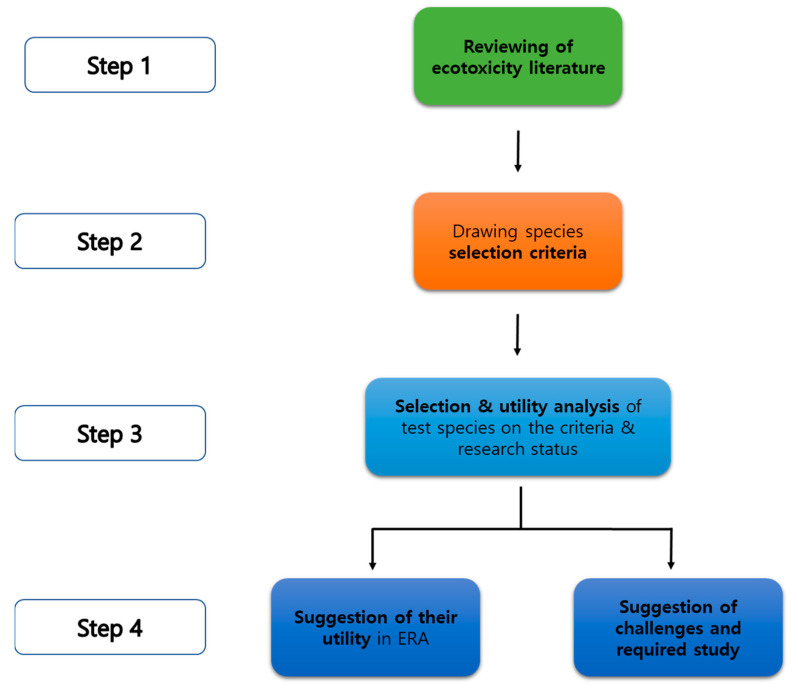
The schematic framework for the ecotoxicity test species selection and utility analysis.

By summarizing existing literature, 9 selection criteria are chosen as follows: (1) clear taxonomy, (2) geological distribution in East Asia, (3) a role in a trophic level, (4) habitat type, (5) well-analyzed morphology and physiological traits such as life cycle, reproduction pattern, (6) applicability to a laboratory study such as ease of manipulation/culture, small size, short life cycle and others, (7) the specific traits of a species, (8) references for chemical ecotoxicity in diverse biological organization levels, (9) sensitivity to chemical exposure [1,2,3]. Using these selection criteria, promising test species were selected (Figure 2). In total, 40 species were randomly chosen and analyzed. Among them, 5 species were selected as a test species (in preparation).

## 3. Mining Promising Test Species

In this section, the traits of each species are analyzed based on selection criteria, and a state of toxicity researches. Then, applicability/utility of each species in effect assessment of ERA is discussed. 

### 3.1. Life Cycle and Physiological Characteristics

In order to observe the chemical effects in the level of individual or higher, the information for taxonomy, geological distribution, habitat type, a role in a trophic level, physiological traits and so forth of five species were required. Also, to establish proper test conditions of a toxicity test for a specific chemical, with chemical properties, the information of species physiological and ecological traits are necessary. The information of the five species are noted as follows.

*Scenedesmus obliquus* (NCBI taxonomy ID: 3088) are one of the unicellular green s, involved in genus *Scenedesmus* that are distributed in the freshwater and brackish water environment, being found in China, South Korea, Japan and so on [9,10,11,12]. And they have the traits of rapid growth, CO_2_ fixation efficiency, growth in the wastewater (e.g., Harsh environment), ease to manage, lipid accumulation, removing metal, having diverse antioxidant [9,10,13]. *S. obliquus* keep growing at the temperature in a range of 15 °C–40 °C [10]. In 25 °C, 150 μmol/(m^2^·s) light intensity, pH 10, the growth rate of *S. obliquus* showed the highest increase level [10,14]. But in another study, at pH 8, the optimization of a growth rate was reported [10]. *S. obliquus* cells are non-mortal and observed in the form of coenobia (microcolonies) of four cells produced by one parent cells, even though under multiple fission mode of reproduction, 8–16 cells can be produced by a cell. Besides asexual reproduction mode, *S. obliquus* have a sexual mode of cell cycle [14].

Macrophytes, including *Hydrilla verticillata* (NCBI taxonomy ID: 51024), have contributed to the improvement of water quality, sediment stabilization and others [15]. *H. verticillata* play an important role in the freshwater ecosystem through the regulation of the nutrient as well as a carbon cycle. Also, as a primary producer, they acts as a habitat provider and energy supplier for other organisms [15,16]. Their habitats involve temperate and tropical regions, and major habitats are ditches, spring, leks, marshes, rivers, tidal zones, channels, quarries, shallow reservoirs [15,17]. Monoecious type is supposed to be originated from Korea [17,18]. Nowadays, they are distributed in Europe, Asia (e.g., Korea, Japan, China), Central Africa, North America, South America, and others [15,17,19]. In reproduction, they are either monoecious or dioecious and the flowers are unisexual. Their flowers and fruits are produced in May to October [17]. Their reproduction is mainly by fragments of stems, but it also can be reproduced through growth of subterranean tubers and turions (axillary buds), suggesting high level of plasticity [15]. 

Aquatic plants and algae are one of the important components of the ecosystem. Thus, the study for them in response to environmental pollutants have attracted the attention of regulatory authorities such as US-EPA, Korea-MOE. In general, among freshwater plants and algae, microalgae are selected as a toxicity test species, since their high sensitivity to municipal and industrial wastewater, easy manipulation in a laboratory. Thus, OECD test guideline 201 and ISO 8692 test guideline for microalgae were established [20]. Moreover, in the OECD 221, ISO 20079, floating rootless flowering plants such as *Lemna minor* were applied to an ecotoxicological test. However, several toxicants did not cause effects upon microalgae, *L. minor*, because of precipitating properties and others, resulting in raising necessary for a submerged macrophyte (rooted plants) such as *H. verticillata* [20].

*Neocardina denticulata* ssp. including *Neocaridina denticulata sinensis* (Kemp, 1918, NCBI ID: 274643) and *Neocaridina denticulata denticulata* (De Hann, 1844, ID: 436129) are classified as a subgroup of *Neocaridina denticulata* (De Hann, 1844. ID: 274642). These are a member of atyidae and native species to Asia including Korea, China, Taiwan, Vietnam, Japan [21]. Their habitats are described as lentic and lotic waters [22] or ponds, rivers, agricultural canals, mountain streams, reservoirs [23]. They have diverse colors and they eat detritus and micro-organisms upon macrophyte roots, and immersed substances as an omnivore. Their total length is 2–3 cm and carapace length of 6.4–7.8 mm in female [22,24]. In other study, carapace length of female and male were 5.1 ± 1.6 mm and 4.4 ± 1.5 mm, respectively [25]. They can live in diverse environmental conditions involving pH (6.5–8.04), temperature (24–29 °C), oxygen contents (5–7 mg/L), ammonia (0.1–1.9 mg/L), nitrate (0.1–10 mg/L), so they can be tolerant to environmental changes, being world-widely distributed even though they are native to East Asia including Korea, Japan, China, Taiwan [23,24,26]. The diameter of eggs is 0.57–1.19 mm on average. Embryonic development lasts for 15 days at the temperature of 27 °C [21,24]. The hatched larvae has a total length of about 2–3.3 mm on average. The number of larvae produced by a female are 21–51 individuals. After about 60 days, the larvae grows to a juvenile stage (total length: 1.2–1.5 cm) and 15 days later, they reach an adult stage [21,23]. Based on the previous study, from a larvae stage, ovigerous females were found in five months, then they can carry the embryos for approximately 30 days [25]. Through 2–3 hatching events, their larva were released. The maximum life span was about 1.3–1.4 years, and main spawning time is Jun-July [22]. Relative to a field, in the laboratory, a slower rate of the growth of this species was observed [25]. The initial growth rate of male was similar with females but with time passed, males grew more slowly, becoming smaller than females according to the previous study [25]. *N. denticulata* ssp. have advantages such as size, availability, ease of culture, and so on. They showed resistance to bacterial infection [27]. They have a transparent exoskeleton, which make possible to carry out noninvasive monitoring of internal organs and tissues, facilitating the investigation of reproduction, molting. What is more, this trait can provide an advantage of large-scale mutant selection [24]. 

*Zacco platypus* (NCBI taxonomy ID: 80810) that is called freshwater minnow, pale bleak, or pale chub, are freshwater fish and distributed to Asia broadly involving South Korea, North Korea, Japan, China, northern Vietnam. They inhabit in the subtropical climate of 10 °C–22 °C [28]. At an adult stage, commonly they are observed in rivers and streams with rapid water flow, whereas they are not found in deep or stagnant waters. They feed on zooplankton, small crustaceans, macroscopic algae, small fish, and detritus [28]. As benthopelagic fish, they fit to toxicity study for both waterborne as well as precipitating/absorbing chemicals. Maximum total length of them is about 22.5 cm and common length of them is about 13 cm. Based on an estimation model (life-history tool), generation time is 1.8 years, Age at the first maturity is 1.8 years (1.4–2.3 years), intrinsic rate of increase 2.56 years [28]. They have about 5 years of life span [29].

*Misgurnus anguillicaudatus* (NCBI taxonomy ID: 75329) are found in Korea, China, India, Thailand, Laos, Siberia (Tugur and Amur drainages), Japan, Vietnam, Taiwan, Cambodia [30]. Moreover, they are introduced into Australia, USA, Germany (Rhine), Italy (Ticino drainages, north of Milano), Aral sea basin and others. They are benthic species and their prominent habitats include ditches, rice paddy fields, streams, and mud places, ponds and they can live without water for a short period, hiding into the mud until water can be available [28,30]. As a benthic species, they are subject to chemicals absorbed upon sediment/debris/soil/submerged plant (e.g., triclosan), having high Koc value and pesticides introduced to rice paddy fields or ditches than other conditions. They live in a subtropical environment. They feed on insects, worms, snails, ostracods, cladocerans, fish eggs, algae, detritus and other small aquatic organisms. Especially, mosquito larvae can be consumed by them [30]. In terms of reproduction, the male *M. anguillicaudatus* can be mature within a year, while female fish can be mature within one or two years [30,31]. Their spawning season is from mid-April to early October and within the period, they spawn multiple times and their fecundity increased in proportion to a body size [30,31]. Female fish yields 1800–15,500 eggs per batch, surviving average 2000 eggs per batch [32]. They inhabit in the subtropical climate of 5 °C–25 °C [28]. Based on an estimation model (life-history tool), their generation time is estimated to be 2.8 years and age at first maturity is 2.6 years. Intrinsic rate of increase is 1.8 years. Their common length is about 15 cm in total length, while maximum length reach 28 cm [28]. Their size in an adult stage is no more than 20 cm, so a laboratory test is plausible. Genetically, they have diverse polyploidy population from 2n to 6n [30]. Triploid cell lines were considered to have superior flesh than diploid, attracting industrial attention [33]. 

### 3.2. Toxicity Study Status

*S. obliquus* as microalgae have been studied in diverse biological organization levels and endpoints. For the oxidative stress assessment, *S. obliquus* were exposed to nanomaterial, metals, copper sulfate, herbicide, fungicide, nitrates and wastewater, in which ROS contents, antioxidative enzyme (e.g., CAT, SOD) activity, antioxidant (e.g., GSH) contents, lipid peroxidation contents and other oxidative stress markers were significantly changed, suggesting *S. obliquus* is suitable test species for detecting oxidative stress [11,34,35,36,37,38,39]. But in these cases, the oxidative stress profiles were significantly affected by humic acid and other chemicals co-treatment.

In addition, *S. obliquus* were used to assess photosynthesis disruption by chemicals including nanomaterial, metals, copper sulfate, herbicide, fungicide, nitrates and wastewater [34,35,36,40,41]. The disruption was evidenced with markers such as Fv/Fm decrease, non-photochemical quenching (NPQ) decrease, chlorophyll contents fluctuation, PSII photochemistry inhibition, and electron transport activity and significant changes of photosynthesis genes such as *psbA*, *petF*, *ATPF0C*, and decline of *rsbS* related to Calvin cycle, while in some cases, humic acid addition changed the responses. In metabolome analysis, *S. obliquus* showed that amino acid biosynthesis and energy metabolism were inhibited by exposure of 1-decyl-3-methylimidazolium nitrate ([C_10_min]NO_3_) and 1-dodecyl-3-methylimidazolium nitrate ([C_12_min]NO_3_) [39]. *S. obliquus* exhibited changes of cellular structure in response to nanomaterials, metals, ionic liquids (IL), pharmaceuticals, and wastewater [11,34,36,37,41,42]. The changes included wrinkle outside of an agal cell, enlarged chloroplast/vacuoles, cell membrane breaking, cell membrane permeability changes, mitochondrial membrane potential changes, irregular crests of a cell wall, increase of a starch granule number/size, loosely packed, disrupted thylakoid membranes, nucleoid disruption, and so forth (Table 1). 

In a population level, *S. obliquus* showed a significant growth rate decline in responses to metals, biocide, flue gas, dioxin, nanomaterials, in which the rate decline was accompanied with oxidative stress and photosynthesis disruption. Also, natural organic compound such as humic acid affected the effect occurrence [11,34,35,46].

In addition, quantitative structure-activity relationships (QSAR) was applied to estimate a chemical toxicity level for *S. obliquus* [47]. Wherein for 21 substituted phenols (2,4-Dichlorophenol, etc.) and anilines (2,4,6-trichloroaniline, etc.), QSAR was established based on the EC50, n-octanol/water coefficient, frontier orbital energy gap (ΔE) [47]. 

*H. verticillata* have been studied in diverse biological organization levels and endpoints under a laboratory condition. In an individual level, when *H. verticillata* were exposed to 2-methyl-4-chlorophenoxy acetic acid (MCPA), perchlorate, metals, fluoride, nanoparticle, they showed the effects such as growth inhibition, leaf dead zone increase, brownish green leaf, hard and thin texture of a leaf, brown stem color, decrease of stem thickness, internode distance and others in both mature and young leaves as well as perforation in surface cuticle of young leaves and others [16,48,49,50,51] (Table 2). 

In the sub-individual level, for *H. verticillata*, most of the studies were focused upon photosynthesis toxicity and oxidative stress. In response to MCPA, perchlorate, metals, fluoride, solvent, nanoparticles, PFAAs mixture, *H. verticillata* exhibited photosynthesis disruption, oxidative stress, protein contents decrease, organelle disruption in leaf/stem tissue [16,19,48,49,50,51,52,53,54,55,56,57,58]. Photosynthesis disruption was proved by pigment content decrease, membrane permeability (e.g., leak of electrolytes increase) increase. Oxidative stress was evidenced by significant changes of antioxidant enzyme (e.g., POD, CAT, GR) activity, antioxidant (GSH, ascorbic acid, anthocyanin, non-protein thiol) contents, ROS contents. In addition, by the chemicals, the significant changes of a total protein level, resistance-causing enzymes (PPO and PAL) activity, DNA methylation, nitrification rate, swollen and loose thylakoid lamella, reduced number of starch grains in mature leaves were observed.

**Table 2:** Test condition and endpoints for ecotoxicity test using *Hydrilla verticillata*.

Chemicals (Test Condition)	Alternative Test Method, Field/Lab., Target Tissue and Others	Sub-Organism Level Endpoints	Organism or Higher Level Endpoints	Reference
**2-Methyl-4-chlorophenoxy acetic acid (MCPA)** (10, 100, 500, 1000 μg/L of MCPA, 7 d exposure)	Laboratory test	Histological change (Purple colored leaves, plasmolyzed leaf cell increase); Pigment contents (e.g., total chlorophyll); oxidative stress (e.g., peroxidase (POD) activity)	Growth rate (e.g., ratio of length of dead zone/total leaf length)	[49]
**TiO_2_;** (24 h exposure)	Laboratory test; TiO_2_ (Rutil, anatase, AEROXIDE P25 (20/80% rutile/anatase, bulk (<5 um rutile, a small amount of anatase)	Oxidative stress (e.g., H_2_O_2_ contents, CAT activity)		[19]
**Perchlorate**	Pigment contents (total chlorophyll, Carotene)		Morphological changes (e.g., leaf colour change, texture of leaf)	[48]
**Fluoride** (0, 10, 20, 40 mg/L, 28 d exposure)	Field species + laboratory test	Protein content, chlorophyll content, carbohydrates content, oxidative stress (e.g., guaiacol peroxidase (POD), GSH)	Growth rate	[16]
**Toluene, xylene, ethylbenzene** (0.1, 1, 5, 10, 50, 100 mg/L, 7 d exposure)	Laboratory test	Photosynthetic pigment contents (e.g., Chlorophyll a/b/(a+b)); Oxidative stress (e.g., SOD)		[53]
**Silver nanoparticle** (500 μg/L of AgNPs, and Ag+; 90 d exposure)	Laboratory test; microcosms condition	Pigment contents (e.g., chlorophyll a/b content); in sediment ammonium nitrogen concentration	Nitrification; Amount of nitrospira; nitrosopumllus; At 90 d biomass	[56]
**Copper** (100 μM Cu(NO_3_)_2_ treatment, 24 h exposure)	Laboratory test; omics analysis	Pigment contents change, Lipid composition changes, membrane permeability change		[52]
**Nickel** (5, 10, 15, 20, 40 μM, 21 d exposure)	Laboratory test; target tissues of stem, leaves	Oxdiative stress (e.g., MDA, POD, PAL, PPO); protein content change	Biomass change;	[54]
**Copper** (0, 0.01, 0.05, 0.1 mg/L; 5 d exposure)	Laboratory test; omics analysis; 2D-page analysis; DNA methylation analysis	Oxidative stress (8-OHdG); DNA-methylation; Protein composition change; pigment content change		[55]
**Copper** (0, 0.01, 0.05, 0.1 mg/L Cu, 5 d exposure)	Laboratory test; difference analysis between mature and young leaves	Pigment content change in mature and young leaves; Histological change (e.g., Partially ruptured surface cuticle)		[57]
**12 PFAAs: PFBA, PFPeA, PFHxA, PFHpA, PFOA, PFOS, PFNA, PFDA, PFUnDA, PFDoDA, PFBS, PFHxS** (0, 1, 10, 100 μg/L, 20 d exposure)	Laboratory test; leaves for analysis of oxidative stress and chlorophyll contents	Chlorophyll content, chlorophyll autofluorescence, oxidative stress (e.g., hydrogen peroxide contesnt)	Biomass, relative growth rate	[58]

*N. denticulata* ssp. have been studied in diverse biological organization levels and endpoints. In an individual level, in responses to 3,4-Dichloroaniline (3,4-DCA), PFAAs, metals, biocides, 4-nonly-phenol, pharmaceuticals, *N. denticulata* ssp. showed mortality in a range from 9.36 (8–10.96) μg/L to 454 (418–494) mg/L [59,60,61] (Table 3).

In terms of sub-individual levels of *N. denticulata*, nonylphenol, dipropyl phthalate (DPrP), imidacloprid (IMI), lindane, chlordane, acetaminophen, Cu^2+^ brought about the significant gene expression changes (immune defense, translation, metabolism, ribosomal gene expression, respiration, stress response, molting), endocrine disruption (estradiol, testosterone concentrations changes, incline of vitellogenin-like protein), oxidative stress (SOD and phenoloxidase activity), organ/tissue level changes (reduced heartbeat rate, gill ventilation) with locomotive activity changes [27,62,63,64,66,67,68,69,70].

*Zacco platypus* have been studied in diverse biological organization levels and endpoints. In a laboratory test condition, *Z. platypus* development stages were either a juvenile stage or an adult stage. In a juvenile stage, average length was 5.5 ± 0.3–7.7 ± 1.04 cm and in an adult stage, ave. length was distributed from 10–20 cm. In juvenile fish ave. weight of 6.49 ± 2.92–9 g, while in an adult stage, average weight was 15.72 ± 4.46 g. Commonly, during 2 w–6 m, they are acclimatized prior to a test in a laboratory. Tests were performed in an acute condition within 96 h–14 d, whereas until now a chronic toxicity test was not conducted. In the laboratory test, water temperature was set at about 19–22 °C and pH of water was 6.8–7.4. Research target tissues included liver, muscle, gill, skin, kidney, muscle, brain (Table 4).

They have shown the potential as a promising test species in a sub-organism/organism level. Precisely by exposure of benzo(*a*)pyrene, cadmium, copper, wastewater contaminated with municipal chemicals (Cd, Co, Cr, Mn, Ni), *Z. platypus* showed oxidative stress occurrence (increase of MDA content, CAT and SOD activity), enhanced biotransformation (CYP1A and CPR activation), genetic toxicity (DNA adduct formation, nuclear abnormality), stress responses (metallothionine expression, heat-shock protein 79/90), endocrine disruption (E2 hormone level, GSI), neurotoxicity (AChE activty) [71,72,73,74,76,77,78,79,80,81]. In addition, Ammonia treatment caused decrease of a survival rate and a hatching rate as well as deformed alevins increase [75].

*M. anguillicaudatus* have been studied in diverse biological organization levels and endpoints. For an individual level, mortality was main endpoints in the study using *M. anguillicaudatus*. In response to biocides, wastewater containing polybrominated diphenyl ethers (PBDE) and metals, *M. anguillicaudatus* showed mortality, growth rate inhibition and histopathological change [82,83,84] (Table 5). 

In a sub-individual level, *M. anguillicaudatus* indicated hepatic damage evidenced by transaminase activity decline in liver, swelled shape and arranged loose in hepatocyte by exposure of dichlorvos, wastewater contaminated with PBDE, metals [83,86]. In genetic toxicity analysis, dichlorvos, flufiprole, copper sulfate, mitomycin C and Trichloroethylene caused DNA strand break to the liver cell, chromosomal aberration increase, micronucleus rate increase [33,81,82,86]. In response to progesterone, phenanthrene, 17β-estradiol (E2), 17α-ethinylestradiol (EE2), flufiprole, Venlafaxine (VFX), o-desmethylvenlafaxine (OVFX), glyphosate, H_2_O_2_, *M. anguillicaudatus* exhibited significant changes of endocrine disruption and development process disturbance, oxidative stress, neurotoxicity, cellular mortality, cellular structure changes, apoptosis occurrence [33,82,87,88,89,90,91].

### 3.3. Characteristics Useful for Chemical Toxicity Tests

*S. obliquus* have diverse traits that can be used as markers in ecotoxicity studies. *Scenedesmus* sp. have plenty of diverse metabolites. Astaxanthin is one of the carotenoid compounds acting as an antioxidant, enhancing preventing UV light influence [9]. Mycosporine-like amino acids that were bound to a chromophore play an important role in protecting UV radiation were exploited for cosmetic skin-care products for UV protection [9]. The expression level of these metabolites would act as a marker for chemical-induced oxidative stress or UV effect analysis. *S. obliquus* have the ability to inhabit in wastewater, so that it can be used in toxicity test under municipal wastewater. Moreover, they can be used in removing pollutants such as metals, ammonium, phosphate and by producing oxygen, reducing chemical oxygen demand [92]. These characters mean that the metabolism enzymes of *S. obliquus* for the pollutant biotransformation can be a useful biomarker to metals, ammonium, phosphate contamination [93]. In addition, *S. obliquus* can form a defense colony in response to zooplankton grazing cues and this colony formation is modulated by cadmium exposure and salinity, suggesting that the colony formation can be markers for cadmium exposure and salinity changes [94].

In *N. denticulata* ssp., The information of nuclear and mitochondrial genome is available at http://huilab.sls. Cuhk.edu.hk/Neocaridina (accessed on 1 January 2021)and comparison analysis of core eukaryotic gene mapping approach (CEGMA) dataset for hormonal and developmental pathway exhibited that the genome shows coverage of expected coding sequences [24]. By applying microsatellites on chromatophore encoded genes and annotation for 65,402 uni-genes were conducted [95]. Moreover, the analysis suggests that they didn’t receive extensive rearrangement. Previous study find the genes related with biological process such as development, growth, molting, reproduction and oxidative stress. sesquiterpenoid pathways, degradation pathways [96]. Also, for ecdysteroid biosynthetic pathway, the genes such as spook, phantom (a cytochrome P450 involved in ecdysteroid biosynthesis), disembodied, shadow were present in *N. denticulata*. Moreover, hormonal regulator and signal transducers such as allatostatins (ASTs), methoprene tolerant (Met), retinoid x receptor (RXR), ecdysone receptor (EcR), calponin-like protein (Chd64), FK509-binding protein (FKBP39), broad-complex (Br-C), crustacean hyperglycemic hormone/molt-inhibiting hormone/gonad-inhibiting hormone (CHH/MIH/GIH) genes were found [96]. For peroxiredoxin and triacylglycerol lipase of *N. denticulata sinensis*, analysis of characterization and expression level in diverse organs were performed [68,97].

In diverse previous studies, *Z. platypus* showed useful traits for ERA. Precisely, male fish reveals a body color change around the time of breeding in June every year [98]. Also, at that time, a nuptial organ is found in the mouth, gills, rear fin of male fish. These types of traits can be used as an indicator for sexual differentiation. Also, *Z. platypus* show behavioral traits such as shorter latency in escape, swimming speed increase under predation [99]. If the change of neurotoxicity biomarker such as AChE activity were associated with the locomotive traits, the mechanism of chemicals bringing about neurotoxicity would be understood precisely. For CYP1A, a previous study reported the tissue distribution and amount of it in *Z. platypus* [73]. In 10 tissues, CYP1A mRNA expression was significantly induced in response to β-naphthoflavone and the expression level were in order of Liver > Gill ≈ Kideny ≈ Intestine > Brain ≈ Gonad > Muscle > Skin ≈ Eye ≈ Heart. CYP1A and CPR mRNA expression showed time-dependent fluctuation tendency, where mRNA expression of them reached a maximum level at early exposure (1–4 days) time while as exposure time passed the expression level decreased [77]. 

Several studies investigating traits of *M. anguillicaudatus* showed they are a promising test species. On the analysis of *M. anguillicaudatus* eye, the genes involving sox2, msi1, bmi1 that control the neural stem cell differentiation were found. The eye of *M. anguillicaudatus* have proliferative and pluripotent neural stem cells which supply new cells during development. Here, in the immunohistochemistry analysis, peroxisome proliferator activated receptors (PPARα, γ) were observed, suggesting that PPARs have a role in neurogenesis of *M. anguillicaudatus* eye. These traits would be useful information for a chemical-induced neurotoxicity study [100]. During a drought, *M. anguillicaudatus* put itself into mud and they can live for weeks or months without water. Under an air-breathing condition, an ammonia concentration in intracellular space would increase, so for survival, *M. anguillicaudatus* have several survival strategies including reducing ammonia production by reducing protein/amino acid catabolism, detoxifying ammonia to glutamine, reducing ammonia production by leading to alanine formation and others [101]. These tolerance traits of them to reducing ammonia contents by diverse molecular mechanisms could be applied to assessing ammonia effect upon target-aquatic environment. 

## 4. Applicability of 5 Species to ERA

In the environment media, by physicochemical properties of chemicals, there are different chemical fates and behavior and this difference can cause different chemical effects on organisms. For example, chemicals easily absorbed to sediment would be more threatened to benthic organisms than pelagic organisms in the aquatic environment. Moreover, By biomagnification through food-chain, the easily bioaccumulated chemicals would be more risk to high level consumer species than producer or primary consumer species. Taken together, in the environment, by diverse factors affecting chemical effects, there can be various environmental chemical exposure scenarios causing different chemical effects on organisms. Thus, to predict precise chemical effects in the environment under a laboratory condition, the application of multi-species having diverse traits covering the chemical environmental exposure scenarios would be reasonable.

This study showed that the traits of five species complement each other in chemical effect assessment on diverse exposure scenarios. *M. anguillicaudatus* live in bottom habitat of water body so they are more suitable in assessing chemical effects having high Koc. *H. verticillata* are sub-merged plants, so they are useful to analyze effects of chemicals precipitating in water than floating plant or algae. As a producer in the food-chain, *S. obliquus* and *H. verticillata* would be sensitive test species in assessing chemicals causing photosynthesis disruption. As a high-level consumer in the food-chain, *Z. platypus* and *M. anguillicaudatus* would be useful in study chemical effects having high Kow than producer or primary consumer. *N. denticulata* ssp. would exhibit chemical effects upon crustacea development and primary consumer.

Moreover, five species are well-studied in life cycle, physiological/ecological characteristics, habitats and they have studied in assessing effects for diverse chemicals such as metal, PFAS, nanoparticles, pharmaceuticals, biocides, PAH and others under a laboratory condition. The effects were studied in diverse biological level endpoints in sub-individual level (e.g., oxidative stress, photosynthesis disruption, xenobiotic transformation, endocrine disruption, immune disruption), individual level (e.g., mortality, reproduction, development, behavior change), population level (e.g., population growth rate) (Table 6). 

In summary, the collective application of the species would be useful in predicting diverse chemical effects upon the East Asia ecosystem under studies in diverse chemical exposure scenarios (Figure 2).

## 5. Remaining Issues in the Study of Test Species

In this section, current ecotoxicity test trends focusing on alternative approaches for regulatory purposes are analyzed. In each paragraph, by diverse alternative approaches, emerging issues/topics are introduced. Then, to increase their utility/applicability in ERA, further study directions for the test species are discussed.

### 5.1. Alternative Testing Methods

Due to rising attention for animal welfare, increase of chemical toxicity information and development of new analysis technology, the requirement for alternative methods reducing/eliminating animal (vertebrate) use was augmented [2,102,103]. In an aquatic toxicity study, the predominant application of a fish toxicity test for a regulatory purpose was reported, relative to other species [104]. Indeed, the UK annual National Statistics report exhibits that fish tests are the most frequently performed tests in non-mammal (birds, reptiles, amphibians, rats, fishes) tests and the fish test number (15%) take second place next to mice (54%), being followed by birds (14%), rats (11%) [105]. Fish and mammal aquatic organisms are classified as vertebrates, so it is necessary to replace/reduce the traditional test as an alternative test. Even though the aquatic invertebrates (*D. magna*), plant (*H. verticillata*) and others are not classified to a vertebrate animal, alternative approaches such as in vitro, in silico, in chemico assay would be helpful to unveil chemical toxicity mechanism in the invertebrates and plants. 

A representative in silico test is the test conducted with a program of quantitative structure activity relationship (QSAR) that is a kind of a mathematical model describing the relationship between chemical structure and biological activity. Using a QSAR program, we can obtain information for biological activity, ADME (absorption, distribution, metabolism and excretion), toxicity of a test chemical without animal sacrifice. It is cost-effective, and rapid relative to in vivo tests and it can be used in filling the data gap in existing toxicity information [106]. In existing literature, there have been diverse trials to develop QSAR. Based on the pEC50 values from *Photobacterium phosporeum* and *Selenastrum capricornutum*, structural features of individual chemicals and their mixture were modeled according to the OECD guidelines. The model was approved to have applicability to predict non-tested chemicals [107]. A numerous endpoint data set for toxicity to algae, Daphnia, fish in response to 3680 chemicals were used to develop a model following OECD principles [108]. But, in this study, only one case of a QSAR study was found for *S. obliquus*. By combining the EC50, n-octanol/water coefficient (logP), frontier orbital energy gap (ΔE), the equation for the single toxicity of substituted phenols, anilines, and mixtures were established [47]. Considering interspecies difference, there would be diverse results for the prediction of chemical effects upon the ecosystem, so the application of test species having diverse traits for developing of QSAR is required.

An in vitro test can be used not only as an alternative to in vivo tests but also, as a screening tool for potential hazard of chemicals [109]. Relative to an in vivo test, an in vitro test has several advantages such as being cost-effective, simple performance, less time consuming, small scale, suitability for evaluating mode of action/for high-throughput test, satisfying ethics requirements and efficiency [109,110]. Thereby, for regulatory purposes, the submission of in vitro test reports is permitted under the regulation of the EU-REACH program. 

A fish or invertebrate embryo has been regarded to be one of practical in vitro systems. Fish in an embryo stage is not classified as a vertebrate animal by NIH [111]. For this, fish embryo toxicity tests have been recommended as an alternative test method. In a comparative ecotoxicity study, fish embryo, *D. magna* or Alga were assessed for 233 chemical compounds, in which lowest-observed effect concentrations (LOECs) was obtained. Therein, the early life stage test based on OECD test guideline 210 indicated 10-fold higher sensitivity of a fish embryo than other systems, suggesting that in some modes of action (MOA) by chemicals, a fish embryo system could be a sensitive tool for detecting chemical toxicity [112]. However, a fish embryo test has some problems. First, a fish embryo test has the lack of endpoints for unveiling the sublethal chemical effects. Second, in some chemical treatments, a fish embryo test showed lower sensitivity than an adult fish test [113,114]. Thus, a previous study suggests the markers such as snout-vent length (≥14% length reduction) and pericardial area (≥3.54 fold pericardial area increases) as an alternative marker. The markers in a fish embryo were predictive of mortality, indicating it would be available as the alternative to a mortality test [114]. In this study, the toxicity test using embryos of *Z. platypus* and *M. anguillicaudatus* were not found to our best knowledge, Thus, considering the advantages mentioned above, the effort to characterize embryos of the species, develop/validate test methods, apply embryo systems to toxicity assessments, and evaluate the sensitivity of embryo systems relative to other species are necessary to increase utility of test species.

Since an invertebrate such as crustacea is not classified as a vertebrate animal, an invertebrate embryo test does not attract the attention relative to a vertebrate case. But there are several advantages of an embryo toxicity test such as utility in the understanding of the toxicity mechanism to development process, reproduction and analysis of chemical effects upon life cycle of test species, so profound studies for it need. In fact, previous studies showed that an invertebrate embryo system could be used to assess chemical toxicity. Embryos of *Marisa cornuarietis* were found to be a suitable system for evaluate endocrine disruption, development toxicity by chemicals. And sensitivity of the embryos is equal or higher than zebrafish embryos [115]. But in this work, no studies using an embryo system of *N. denticulata* ssp. are found. 

Cellular systems including primary cell, cell lines, stem cell have been used as an important alternative tool in ecotoxicity test for environmental chemicals. In fish and shellfish, numerous studies using primary cells showed it is a useful system to assess chemical ecotoxicity such as endocrine disruption, oxidative stress, apoptosis and immune disruption [116,117,118,119]. Cell lines also have been applied to an in vitro toxicity test [104]. So far, several cell lines including RTL-W1, RTgill-W1, RGT2 of *Oncorhynchus mykiss*, PLHC-1 of *Poeciliopsis lucida*, and CHSE-14 of *Oncorhynchus tshawytscha* have been applied to an ecotoxicity test, which includes [104]. Cell lines have advantages including homogeneity, ease to culture, and being suitable for a high-throughput test. A cytotoxicity test using a gill cell line of *O. mykiss* indicated an approximately 1:1 correlation with a fish acute toxicity test result in responses to organic chemicals [120]. Interlaboratory reproducibility of a test method using a fish gill cell line (RTgill-W1 cell line) was demonstrated [121]. This suggests that fish gill cell lines can be a useful alternative tool for in vivo toxicity. Additionally, a stem cell system has received attention because of useful traits. It has characteristics such as self-renewal, differentiation to other cell types, forming tissue originated from the same cell, which are not found in cell line and primary cell systems. Therefore, the problems including availability limitation, functional variability, genetic alterations could be eased [65]. 

In terms of this work, in vitro tests using a cell line, primary cell, stem cell, embryos were not detected in these species except for a cellular level study for *M. anguillicaudatus*. As mentioned above, those systems have diverse advantages in chemical ecotoxicity assessment and the application of the systems is encouraged in future ecotoxicity tests. Thus, not only development, but also applicability the evaluation of the systems originated from a test species would be helpful for elevating the species utility.

### 5.2. Other Alternative Approaches

Omics is a discipline of biology that deals with genomics, proteomics, transcriptomics, metabolomics [122]. Since omics is based on total information of the gene, transcript, protein, or metabolite produced in an organism, the information on it can enhance the understanding of total response profiles to chemical exposure [123]. For example, under PFOS exposure, analysis using omics technology presented neurological function change, oxidative stress, energy metabolism disruption, axonal deformation, neuroinflammatory stimulation, calcium ion signaling dysregulation, suggesting that omics analysis can be a tool to broaden understanding of a toxicity mechanism [124]. However, in this review, a few case studies evaluating chemical toxicity using omics technology were found in terms of *S. obliquus*, *H. verticillata*, *N. denticulata* spp., whereas no study exist for *Z. platypus*, *M. anguillicaudatus*. National Center for Biotechnology Information (NCBI) database showed that whole genome sequence of *M. anguillicaudatus* (assembly ID: HAU_Mang_1.0/GCA_027580225.1 (reference genome)) and *S. obliquus* (7 assemblies including UMN_S.PABB004_v1) are published, suggesting that they can be used in studies such as RNA sequencing analysis and other omics technologies for unveiling chemical toxicity mechanism [125]. 

When using omics technology, mining of diverse genes and proteins useful for chemical effect assessment can be possible. Among those, the genes relevant with xenobiotic biotransformation, development, endocrine control, reproduction, immune system as well as house-keeping genes are important in the understanding of chemical effect. Xenobiotic biotransformation is the process that transform lipophilic chemicals to hydrophilic chemicals, eliminating them out of body. In model species such as *Danio rerio*, genes and enzymes related with xenobiotics biotransformation are well evaluated [126]. But, in the five species of this work, lack of information for the genes was found. For example, in case of *Z. platypus*, cytochrome P450 systems are characterized, whereas the characterization of cytochrome P450 systems of *M. anguillicaudatus* and *N. denticulata* spp. was not reported [73,77]. To detect a background level within an organism, in case of transcript/translation analysis, housekeeping (reference) gene/protein expression levels are used, since their expression are maintained at a constant level in most of the organs and the expression level cannot be changed significantly [127]. In case of plant, *Arabidopsis pumila* showed about 10 genes that were recommended as reference genes by specific stress conditions. The application of UBQ9 (Polyubiquitin 9) and GAPDH (Glyceraldehyde-3-phosphate dehydrogenase) in heat stress, ACT1 (Actin 1) and GAPDH in salt stress, UBC35 (Ubiquitin conjugating enzyme 35) and GAPDH in cold stress, ACT1 and GAPDH in comparison with different tissues were suggested [128]. In *D. magna*, exposed to ibuprofen, GAPDH, UBC (Ubiquitin conjugating enzyme) showed stability relative to other candidates such as 18s, 28s genes [129]. Among the species reviewed in this work, well-known housekeeping genes such as β-actine (ACTB), elongation factor 1 alpha (EF-1a), GAPDH, 18S ribosomal RNA (18S rRNA) were analyzed and expression profiles in terms of gender difference, tissue type, different developmental stages were studied in *Misgurnus anguillicaudatus* [85]. *Z. platypus* beta-actin gene expression was used as a reference gene and protein [77,79]. In *N. denticulata*, beta-actin was used as an internal control [62]. This suggests that the identification/evaluation of the reference genes under a different condition is required. By extension, the genes related to endocrine system, reproduction, development, immune system and others can be significantly related with adverse outcome [2]. Thus, profound study for searching the genes using omics technology are required. 

Most of dossiers submitted to regulatory authorities (e.g., EU-ECHA) for proving chemical safety are mainly composed of in vivo test reports performed at the individual or higher level [130]. The reason for this is that relative to an in vitro test, an in vivo test is considered to have higher ecological relevance [104]. Adverse outcome pathway (AOP) is a conceptual framework composed of molecular initiation events, adverse outcome [2]. It can be used for the prediction of adverse outcome in an individual or higher level by using the information for molecular level events. For this, AOP could augment trustworthy/utility of in vitro test, in silico test by increasing ecological relevance, finally reducing animal sacrifice. In this sense, to establish AOP to chemical exposure, the information for chemical effects in diverse biological organization levels is required (Figure 1). Especially, nuclear receptor activation (e.g., estrogen receptor) can act as molecular initiation event (MIE) that play a role as a starting point in AOP, since nuclear receptor activation can trigger cell signaling and physiological changes followed by chemical-induced reproduction disruption, growth rate inhibition and others [2,131,132]. This suggests that chemical toxicity assessment with nuclear receptor activation would be reasonable in establishing AOP. However, this study showed that the toxicity endpoints for five species were concentrated upon specific biological organization levels. For example, the molecular level study for *H. verticillata* were concentrated on oxidative stress and photosynthesis disruption. Also, in five species, little information for molecular/cellular/organ level responses involving nuclear receptors activation were available. 

The perfect replacement of animal tests with an alternative test is not practicable due to the complexity of a biological system [103]. Rather, an alternative test can provide a piece of information, and thus, the collective application of the results from alternative test (e.g., in vitro test) and in vivo test might be practical to assess risk of chemicals. For these reasons, integrated approaches to testing and assessment (IATA) by OECD, accelerating pace of chemical risk assessment (APCRA), EU ToxRisk are developed [103,133]. Among them, IATA is a type of method for characterizing chemical hazard and assessing chemical safety by integrating multiple sources of information and producing new data for regulatory decision-making about chemical hazard/risk [134]. This suggests that the application of diverse research results using the test species based on alternative test methods as well as conventional methods would increase utility of test species for regulatory purposes.

### 5.3. Traits Specific Issues to Given Species

The specific markers found in a specific test species can influence test species utility. For example, male fish of *Z. platypus* showed a nuptial organ on their mouth, gills, rear fin. Changes of these traits can be a marker of endocrine disruption, sexual development. Also, the molecular level events for development of the organ would be used as a biomarker showing chemical induced developmental effects. *M. anguillicaudatus* show tolerance to ammonia exposure by suppression of amino acid metabolism. *H. verticillata* can mitigate the metal contamination by absorbing it through their root. These properties mean that the molecular level changes to the ammonia, metals exposure could be a sensitive early-warning signal. *S. obliquus* have a range of metabolites such as astaxanthin that can be used as a biomarker for oxidative stress and they inhabit in wastewater, so they can be cultivated/tested in municipal wastewater growth medium. *S. obliquus* form a defense colony when they are exposed to zooplankton grazing cues, while this system is impaired by cadmium exposure and a salinity change. These traits indicate that an integrity of defense colony system of *S. obliquus* can be a marker suggesting cadmium exposure and salinity increase [94]. Additionally, the male *G. holbrooki* size works as an advantage in mating success with female, paternity, sperm quantity, sperm quality, suggesting that change profiles of male fish size could be used as a reproduction toxicity marker [135].

Through comparison of test species with model species, utility/potential of them can be proved in toxicity research area in which the utility of model species is well-evaluated. *P. ramosa* showed higher sensitivity to six chemicals, when they are compared to *D. magna* [136]. *D. similis* involved in the same genus with *D. magna* showed significantly different response profiles to environmental chemical exposure with *D. magna*, suggesting their utility relative to *D. magna* [137]. CYP450 1A gene expression of *Z. platypus* gills was compared to the expression of gills of *O. latipes*, *C. carpio*, *D. rerio* in response to benzo(*a*)pyrene. Wherein, highest sensitivity was observed in the *Z. platypus* gills. *N. denticulata* showed more sensitive responses to pain relief drugs such as acetaminophen and biocide dichlorooctylisothiazolinone exposure than *D. japonica*. Following glyphosate exposure, *H. verticillata* showed a significant increase of oxidative stress and pigment contents, while *V. natans* don’t show reverse responses/no responses [138]. By triclosan, *M. anguillicaudatus* showed the most sensitive response in terms of acute mortality compared to insects (*C. plumosus*), shrimp (*N. denticulata sinensis*), *Daphnia magna*, *P. parva*, Annelids (*L. hoffmeisteri*), *R. Limnocharis* [84]. *M. anguillicaudatus* showed higher tolerance to ammonia exposure than *P. parva*, *A. liaoningensis*, *C. giurinus*. *S. obliquus* showed a higher ammonia removal rate than *Chlorella vulgaris*. Considering utility of model species in an ecotoxicity study, this type of comparison can be an effective approach to improve test species utility in ERA. 

Prior to a comparative study, the definition of the model species is important prerequisite step. Thus, by extension, in this paragraph, this manuscript presents several model ecotoxicity test species enrolled in international test guideline focusing OECD and EPA test guidelines. On the OECD test guidelines, recommended fish involve *Danio rerio* (OECD TG 203, 212, 215, 236, 229), *O. mykiss* (OECD TG 203, 212, 215), *Cyprinus carpio* (OECD TG 203, 212), *Oryzias latipes* (OECD TG 203, 212, 215, 240, 229), *Pimephales promelas* (OECD TG 203, 212, 229). Also, as a supplementary species, *Carassius auratus*, *Leopomis macrochirus*, *Menidia peninsulae*, *Clupea harengus*, *Gadus morhua*, *Cyprinodon variegatus* (OECD TG 212). In terms of invertebrates, Daphnia genus such as *Daphnia magna*, *Daphnia pulex* and *D. japonica* were recommended (OECD TG 202/211). In freshwater alga and cyanobacteria, *Pseudokirchneriella subcapitata* and *Desmodesmus subspicatus*, *Navicula selliculosa*, *Anabaena flos-aquae* (cyanobacteria), *Synechococcus leopoliensis* (cyanobacteria), (OECD, TG 201). Freshwater aquatic plant, genus *Lemna* (duckweed, OECD, 221). Freshwater dipteran *Chironomus* sp. (*C. riparius*, *C. tentans*). In other aquatic organisms, *Lymnaea stagnalis* (OECD, 243), *Xenopus laevis* (OECD, 2009).

By the US-EPA aquatic toxicity methods, in invertebrates, *Daphnia magna*, *Daphnia pulex*, *Ceriodaphnia dubia*, and as a shrimp-like crustaceans, Mysids (*Mysidopsis bahia*, *Holmesimysis costata*), Brine shrimp (*Artemia salina*) were listed in the test guideline. For a fish, *Pimephales promelas*, *O. mykiss*, *Salvelinus fontinalis*, *Cyprinodon variegatus*, Silversides: inland silverside (*Mendia beryllina*), Atlantic silverside (*Mendia menidia*), Tidewater silverside (*M. Peninsulae*) were listed (US-EPA, 2002). In addition to the species listed above, as a supplemental organism, freshwater vertebrates living in warmwater including *Cyprinella leedsi*, *Lepomis macrochirus*, *Ictalurus punctatus*, freshwater invertebrates living in cold water including *Pteronarcys* spp., *Pacifastacus leniusculus*, *Baetis* spp., *Ephemerella* spp., freshwater invertebrate living in warmwater including *Hyalella* spp., *Gammarus lacustris*, *G. fasciatus*, *G. pseudolimnaeus*, *Hexagenia limbate*, *H. bilineata*, *Chironomus* spp. were in test species list of EPA-guideline. The precise information for life span, culture and toxicity test methods of each species is well-noted in each test guideline [7,139] and other brief information such as species identity and whole genome information are summarized in Table 7.

### 5.4. Ecological Relevance 

To predict environmental chemical effects upon the ecosystem in a laboratory, conducting an ecotoxicity study under ecological conditions is required. Indeed, numerous studies showed that there was a significant change of chemical effects under ecological conditions such as chemical mixture treatment, multi/trans-generation, coexistence with predator/prey, flow-through exposure and others. For example, PFOS exposure with pentachlorophenol inhibited growth of *S. obliquus*, while atrazine and diuron increased the growth, suggesting that coexistence of other chemicals can cause chemical toxicity changes [44]. By the chemical exposure through multi-generations, different effects such as intersex occurrence relative to one generation chemical exposure condition were caused in an offspring [140,141]. Under the co-existent condition with a predator, *Z. platypus* showed a behavioral change such as swimming speed increase and shorter latency in case of an escape response [99]. Similarly, with predators, *D. similis* showed inducible morphological defenses [142]. Under the competition with *R. raciborskii*, *S. obliquus* showed increase of oxidative stress [38]. With the food (*Rseudokirchneriella subcapitata*), 48 h-acute toxicity of *D. similis* was suppressed [143]. These findings suggest that co-existence of predator, competitor, prey could induce changes of chemical ecotoxicity. However, a few case toxicity studies were found on ecological conditions using *S obliquus*, *N. denticulata* (Table 1 and Table 3). 

In other cases, the applicability of test species in chemical effect prediction can be evaluated in a field, microcosm, mesocosm condition. In the study using *H. verticillata*, instead of a field study, the conditions such as microcosm and mesocosm that mimic the ecosystem, were applied to assess chemical toxicity [54]. In addition, in the field contaminated with perchlorate, *H. verticillata* showed diverse decay in leaf, and stem (Table 2). In the study using *M. anguillicaudatus*, the effects of wastewater under the condition that they were caged in a field were analyzed (Table 5). By contrast, in case of *Z. platypus*, field and laboratory test are carried out in diverse chemical effect analyses. Indeed, biomarkers related to oxidative stress, genetic toxicity, biotransformation that were assessed for metal exposure in a laboratory condition are proved to be applicable in a field (Table 4) [74,77,78,79]. 

Also, to predict chemical effects upon the ecosystem, species habitat type should be considered. Test species ecological traits such as a habitat type (e.g., benthic/pelagic habitat of aquatic animals and submerged habitat/water surface habitat of aquatic plants/algea) can cause different responses to chemicals exposure. Submerged plant and benthic species can be easily exposed to chemicals that are precipitated or absorbed to sediment, whereas floating/waterborne chemicals are more hazardous to pelagic species or floating plants/algea. As such, this review suggests benthic species *M. anguillicaudatus* and submerged plant *H. verticillata* with benthopelagic species *Z. platypus* and floating algea, *S. obliquus*. However, no comparison studies considering chemical fate and behavior in an environmental medium by physico-chemical properties (e.g., Koc, Kow, solubility) were observed in the collective application of them.

### 5.5. Standadization of Recommended Species 

Finally, to develop or enroll these test species in international test guidelines, establishing a standard culture methods in laboratory and standardizing the recommended species should follow. Also, to update the diverse international test guidelines, proposing new test draft guideline, discussing and proofreading of the draft, meeting with international experts relevant to the field of ecotoxicity testing, and interlaboratory cross-checking the draft guidelines should be carried out in the future.

## 6. Conclusions

Based on the test species selection criteria in existing literature, *S. obliquus*, *N. denticulata*, *Z. platypus*, *M. anguillicaudatus*, *H. verticillata* distributed in East Asia are suggested as promising test species. As an analysis result for them, this study showed that their utility in ERA varied by their physiological/ecological traits and a research state, suggesting that there would be no species fit for all ERA objectives, so multiple species application to ERA is necessary. Especially, even though five species exhibit the overlapped geological distribution, they have differences in habitat, ecological traits (e.g., trophic level, habitat type), physiological traits (e.g., life cycle, sensitivity to chemicals), a state of an ecotoxicity study. Thus, five species can be a battery in chemical effect assessment under diverse exposure scenarios.

By analyzing recent ecotoxicity test trends, this review shows that alternative approaches are important emerging issues in a study for increase of test species utility in ERA. In detail, test species application to QSAR development, cellular system development, conducting toxicity test using embryos are suggested as further study issues. This work shows that using omics technology, mining housekeeping genes and biomarker genes relevant with xenobiotic biotransformation, endocrine disruption, and neurotoxicity would be future study areas. Also, for the development of AOP, chemical effect analysis in various biological organization levels is suggested as another further study issue for utility increase of test species. 

As a non-alternative approach, a comparison study to find interspecies difference (e.g., sensitivity), development of markers specific to a test organism, ecotoxicity test under ecological conditions, and others are suggested as the remaining issues. Accordingly, an integrative study of data from diverse alternative approaches as well as conventional ecotoxicity test would assist in test species utility increase.

The utility information of five species and further study issues discussed in this review would be helpful not only in the application of the five species to ERA, but also in mining a promising test species and increasing their utility in ERA, resulting in effective performance of ERA for regulatory purposes.

## Figures and Tables

**Table 1:** Test condition and endpoints for ecotoxicity test using *Scenedesmus obliquus*.

Chemicals (Test Condition)	Alternative Test Method, Field/Lab., Target Tissue and Others	Sub-Organism Level Endpoints	Organism or Higher Level Endpoints	Reference
**ZnO** (0.25, 0.5, 1 mg/L, 72 h exposure)	Laboratory test; toxicity under UV/dark/visible light	Cellular malformation (e.g., internalization into cell wall); pigment content; Oxidative stress (ROS contents); Cell membrane disruption (Lactate dehydrogenase contents)	Growth inhibition rate	[43]
**PFOS, and either** **Pentachlorophenol, trazine, or diuron/** (10–40 mg/L PFOS, 10–40 mg/L PFOS, 72 h exposure)	Laboratory test	Oxidative stress (ROS contents)	Growth inhibition; Uptake of pentachlorophenol, atrazine, diuron	[44]
**Graphene oxide (GO), humic acid (HA)/** (5, 10, 20, 40, 80 mg/L GO, 72 h exposure)	Laboratory test; mixture toxicity of GO with/without humic acid	Oxidative stress; cellular morphological change (e.g., cell agglomeration with GO in surface)	Growth rate inhibition of 72 h-LC50	[11]
**AgNp/** (for a growth inhibition test, 0, 10, 50, 100, 1000, 2000 μg/L AgNP; 7 d exposure; for a metabolite analysis, 0, 1, 10, 100 μg/L AgNP, 7 d exposure)	Laboratory test; Omics analysis	metabolite contents change (e.g., d-galactose, sucrose)	Growth inhibition	[45]
**Ionic liquids (e.g., 1-hxyl-3-methylimidazolium nitrate)**/ (0–20 mg/L for all Iionic liquids, 96 h exposure)	Laboratory test	96 h—cell membrane permeability; Cell morphological change; Pigment contents; Photosynthesis rate (Fv/Fm, Y(II), NPQ)		[41]
**Non-steroidal anti-inflammatory drugs (NSAIDs): Ibuprofen (rac-IBU, S-(+)-IBU), aspirin (ASA), ketoprofen (KEP)**/ (for rac-IBU, S-(+)-IBU, ASA 1–300 mg/L; for KEP 0.01~12 mg/L; 96 h exposure)	Laboratory test; isomer toxicity analysis	Cell morphological change using TEM analysis (e.g., turgid, plasmolysis, irregular peaks); pigment content; photosynthesis activity; photosynthic-electron transport related gene expression level (e.g., *PsaA*, *PsaB*)	Growth inhibition; Photosynthetic rate; Respiratory rate	[42]
**Flue gas component (CO_2_, NO, SO_2_, NaHSO_3_, pH), temperature** (for NO, 5, 10, 15% for 9 d, for SO_2_ 0, 100, 200, 300, 500 ppm, 72 h, for NaHSO_2_: 0, 50, 100 ppm, 72 h)	Field species + laboratory test; Flue gas, pH, temperature effect analysis		Growth inhibition; removal efficiencies; recovery capability	[46]
**Wastewater**	Laboratory test; wastewater effect depending on treatment process	Pigment contents; cell morphological change (membrane integrity)	Growth inhibition	[34]
**21 substituted phenols and anilines**	Laboratory test; QSAR estimation		QSAR estimation	[47]
**Sanguinarine** -10, 20, 40, 80 μg/L, 96 h exposure		Photosynthesis activity; oxidative stress	Growth rate inhibition	[38]
**TiO_2_** (10, 50, 200 nm sizes of 10 mg/L of TiO_2_, 72 h exposure)	Laboratory test	Cellular morphological change (e.g., wrinkle outside of agal cell,); Photosynthesis activity (NPQ, Fv/Fm); oxidative stress (ROS contents); Pigment content	Growth inhibition; oxygen evaluation; oxygen respiration	[36]
**Lactofen, desethyl lactofen, Acifluorfen** (3, 2.5, 2, 1.5, 1, 0.5, 1 μg/L of S-lactofen, 3, 2, 1.5, 1, 0.7, 0.5 μg/L of rac-lactofen, 2, 1.5, 1, 0.7, 0.5, 0.3 μg/L of R-lactofen, 3, 1.5, 0.75, 0.5, 0.25, 0.1 μg/L of desethyl lactofen, 1, 0.5, 0.25, 0.15, 0.075, 0.04, 0.02 μg/L of acifluorfen)	Laboratory test; isomer toxicity analysis	Oxidative stress; pigment contents	Growth inhibition of 96 h-EC50	[35]
**Silver nanocluster, Silver ion, L-cystein** (0, 33.75, 67.5, 135, 270, 540 μg/L (silver atom based), L-cystein used to chelate Ag+, 5, 10, 20 μg/L of Ag+; AgNC (0, 135 μg/L) + L-cysteine (0.5 mM), 96 h exposure)	Laboratory test; Omics analysis (transcriptome analysis)	Photosynthesis-electron transport related gene expression level (e.g., *PsaA*); pigment contents; RNA-sequencing (calvin cycle, light reaction of photosynthesis related gene significantly disrupted)		[40]
**1-decyl-3-methylimidazolium nitrate ([C_10_min]NO_3_), 1-dodecyl-3-methylimidazolium nitrate ([C_12_min]NO_3_)** (0.01, 0.5 mg/L for NO_3_ and 0.0005, 0.001, 0.005, 0.01, 0.02, 0.08, 0.3, 0.5, 0.,8 mg/L for NO_3_)	Laboratory test, omics (metabolomics)	Oxidative stress (e.g., ROS contents), metaolomic analysis using GC-MS, light quantum yield (Y(II)), electron transfer rate (ETR)	Growth inhibition	[39]

**Table 3:** Test condition and endpoints for ecotoxicity test using *Neocaridina denticulata* ssp.

Chemicals (Test Condition)	Alternative Test Method, Field/Lab., Target Tissue and Others	Sub-Organism Level Endpoints	Organism or Higher Level Endpoints	Reference
**4-Nonylphenol (4-NP)** (0.001, 0.01, 0.1, 0.5 mg/L)	Laboratory test; omics analysis; analysis of expressed sequence tags; semi-quantitative mRNA; flow-through exposure	Transcription level (levels, *Hemocyanin*, *elongation factor 1-alpha*)		[62]
**4-Nonylphenol (4-NP)**	Field species + Laboratory test; interspecies difference test (*Dugesia japonica*, *Physa acuta*, *Ceridaphnia cornuta*, *Caridina pseudodenticulata*, *Mona macrocopa*)		Mortality (96 h-LC50, NOAEL)	[63]
**3,4-Dichloroaniline** (0.625, 1.25, 2.5, 5, 10 mg/L; 96 h exposure)	Field organism + laboratory test		Mortality of 96-LC50	[61]
**Dipropyl phthalate (DPrP)** (1, 5, 10, 50 mg/L, 10 d exposure)	Laboratory test	Immune toxicity (acid phosphatase activity, α-naphthyl acetate esterase, β-glucuronidaseactivity, phenoloxidase activity, superoxide dismutase activity, haemocyanin mRNA		[27]
**Acetaminophen(APAP), Ibuprofen (IBU)** (0.2, 0.6, 1, 1.5, 2, 4, 8 mg/L)	Laboratory test; mixture toxicity of APAP and IBU in 3 combinations		Mortality of 96 h-LC50	[60]
**Imidacloprid (IMI)** (0.03125, 0.0625, 0.125, 0.25, 0.5, 1 mg/L, 96 h exposure)	Laboratory test; target organ: heart, gills; locomotive activity change according to co exposure of acetylcholine or not	Organ toxicity (e.g., heart beat rate, gill ventilation rate); Oxidative stress (e.g., ROS content, 4-hydroxynonenal); energy metabolism (e.g., Glucose contents)	by IMI only 72 h-Locomotor activity-; by IMI with acetylcholine locomotor activity+	[64]
**10 biocides (e.g., methylisothiazolinone**	Laboratory test; 10 chemical toxicity difference		Mortality of 96 h-LC50	[65]
**Acetaminophen (ACE),** **Aspirin (ASP), Diclofena (DIC), Ibuprofen (IBU), Mefenamic acid (MFA), Naproxen (NAP)** (0, 0.001, 0.01, 0.1, 1, 10, 100 mg/L ACE; 0, 0.0001, 0.001, 0.01, 1, 10 mg/L for ASP, DIC, IBU, MFA, NAP)	Laboratory test	Neuronal system disruption (e.g., Cholinesterase activity); Energy prifile (ATPase activity)		[66]
**Chlordane, Lindane,** **17β-estradiol** (for Chlordane: 0.005, 0.01 0.03, 0., 0.3 μg/L, for Lindane, 0, 1, 3, 5, 10, 20, 30 μg/L)	Field species + laboratory test	Endocrine disruption (testosterone level)		[59]
**Chlordane, Lindane,** **17β-estradiol** (for Chlordane: 1 and 10 ng/L; for Lindane, 0.1 and 1 μg/L, 28 d exposure)	Field species + laboratory test	Endocrine disruption (estradiol level)		[67]
**Mn^2+^, Ba^2+^, Cu^2+^, Mg^2+^, Ca^2+^, Zn^2+^, K^+^** (10 mM or 1 mM for each chemical)	Laboratory test, target tissue (Intestine, hepatopancreas, muscle, testis, ovary, gill, epidermis, heart, eyestalk)	Peroxiredoxin gene expression, enzyme activity		[68]
**Cu^2+^** (2.5 μmol/L, 48 h exposure)	Laboratory test; target tissue (cephalothorax), omics analysis for transcriptome	Gene expression change of *transglutaminase 2*, *programed cell death protein 7-like* and others		[69]

**Table 4:** Test condition and endpoints for ecotoxicity test using *Zacco platypus*.

Chemicals (Test Condition)	Alternative Test Method, Field/Lab., Target Tissue and Others	Sub-Organism Level Endpoints	Organism or Higher Level Endpoints	Reference
**Field water**	Field test; target tissue/organ: liver, blood, spleen, gonad,	Biotransformation activity (EROD); genetic toxicity (DNA strand breakage); neuronal system disruption (AChE activity), endocrine disruption (VTG expression), liver toxicity (alanine aminotransferase), serum macromolecule content (total cholesterol, protein content, creatine)	Gross indices (CF=, LSI+, VSI(viscera somatic index)+, SSI (spleen somatic index)+, Population health responses; reproductive toxicity (e.g., fecundity, oocyte diameter)	[71]
**Contaminant containing metal** (Cr, Cu, Zn, Cd, Pb, Hg)	Field test	Oxidative stress (e.g., CAT); stress protein (metallothionein)	Gorss indices (e.g., CF, LSI)	[72]
**β-naphthoflavone** (β-naphthoflavone 1 μM, 96 h exposure)	Field species + laboratory test; target tissue/organ: liver, gills, kidney, intestine, brain, gonad, muscle, skin, eye, heart; CYP1A cDNA sequencing	Biotransformation (*CYP1A*)		[73]
**Environmental contaminants**	Field test; target tissue/organ of liver, kidney, gill	Histological analysis (Degree of tissue change); Oxidative stress	IBR (integrated biomarker response)	[74]
**Ammonium Chloride** (10, 50, 100, 200, 500 mg/L)	Field species + laboratory test		Survival rate; Reproductive toxicity(hatching rate); Deformed alevins	[75]
**Cadmium chloride** (3, 30, 300 μg/L, 49 h exposure)	Laboratory test	Stress protein (metallothionein)		[76]
**Benzo(*a*)pyrene** (4, 20, 100 μg/L, 14 d exposure)	Laboratory test; target tissue/organ of liver	Genetic toxicity (DNA adduct Content); biotransformation (CYP1A expression)	Gross indices (HSI, GSI, CF)	[77]
**Field water**	Field test; analysis of seasonal marker changes in field	Relative to up-stream, Hormone level at may Endocrine disruption (intersex) Biotranformation (EROD); neuronal system disruption (AChE)	Gross indices Relative to up-stream, Size distribution	[78]
**Benzo(*a*)pyrene** (4, 20, 100 μg/L, 14 d exposure)	Laboratory test; target tissue/organ of liver, gills; interspecies difference analysis (*Zacco platypus*, *O. latipes*, *D. rerio*, *C. carpio*)	Biotransformation (*CYP1A* expression in gills and liver)	Gross indices (CF, LSI, GSI)	[79]
**Environmental pollutants from municipal region**	Field test; target tissue/organ of liver, gills;	Gene and protein expression of *HSP 70/90*, *SOD*, *CAT* and stress protein (e.g., metallothionein)		[80]
**Environmental pollutant** (mitomycin C (0, 0.2, 2.0, 20, 200 μg/L); Trichloroethylene (0~3000 μg/L))	Field/in housed species + Laboratory test; interspecies difference analysis (*Zacco platypus*, *Carassius* sp., *Misgurnus anguillicaudatus*, *Odontobutis obscura obscura*, *C. carpio*, *R. ocellatus ocellatus*, *Leiognathus nuchalis*, *Ditrema temminchki*)	Genetic toxicity marker (e.g., chromosomal aberrations)		[81]

**Table 5:** Test condition and endpoints for ecotoxicity test using *Misgurnus anguilicaudatus*.

Chemicals (Test Condition)	Alternative Test Method, Field/Lab., Target Tissue and Others	Sub-Organism Level Endpoints	Organism or Higher Level Endpoints	Reference
**Imidacloprid** (for acute test, 115, 132.25, 152.09, 174.90, 201.12 mg/L 96 h exposure; for biomarker 43, 67, 91, 115 mg/L, 6 d exposure)	Field species + laboratory test; target tissue/organ of liver testis, blood	Histopathological change (e.g., testis:disorganization) genetic toxicity (e.g., erythrocyte micronuclei assay) Liver toxicity	Mortality: LC50-96 h	[85]
**Dichlorvos (DDVP)** (for an acute toxicity test 0, 4.56, 5.76, 7.12, 8.96 μg/L, 96 h exposure; for an transaminase test: 0.64, 1.28, 1.92, 2.56, 3.2 μg/L, 6 d exposure)	Field species + laboratory test; target tissue/organ of liver, serum	Liver toxicity (Transaminase activity); Genetic toxicity (e.g., erythrocyte micronuclei rate, DNA strand breakage)	mortality of 96 h-LC50	[86].
**Progesterone (P4)** (0, 10, 100, 1000 ng/L, 28 d exposure)	Field species + laboratory test; target tissue/organ of liver, kidney, heart, brain, gonad	Dax1 gene transcription level in each organ		[87]
**Glyphosate** (0, 80, 240, 400, 560 mg/L, 24 h exposure)	Laboratory test; cellular system application; diploid and triploid fin cell lines	Cellular viability test (MTT assay); oxidative stress (SOD activity); neuronal system change (AchE activity); genetic toxicity (micronucleus assay)		[88]
**Mitomycin C, Trichloroethylene** (mitomycin C (0, 0.2, 2.0, 20, 200 μg/L); Trichloroethylene (0–3000 μg/L))	Field species + laboratory test; interspecies test (*Carassius* sp., *Zacco platypus*, *Misgurnus anguillicaudatus*, *Odontobutis obscura obscura*;); target organ of gills and serum	Genetic toxicity marker (e.g., chromosomal aberrations)		[81]
**Flufiprole, flufiprole isomer and 6 metabolites** (20, 40, 80 μg/L, 96 h exposure)	Laboratory test; toxicity comparison with isomer and metabolites; target organ of liver, gills, blood	Oxidative stress	Mortality of 96 h-LC50	[82]
**Phenanthrene** (1.26, 1.58, 2, 2.51 mg/L)	Laboratory test; target organ: test organ of liver, testes, ovary, serum; sexual difference to VTG expression	Endocrine disruption (VTG expression)	GSI	[89]
**Triclosan** (for an acute test, 96 h exposure, 0, 0.02, 0.03, 0.044, 0.067, 0.1, 0.15, 0.225 mg/L; for an chronic test, 30 d exposure, test con. 0, 0.003, 0.005, 0.007, 0.01, 0.015, 0.023 mg/L)	Laboratory test; interspecies difference (*P. parva*, *C. auratus*, *M. anguillicaudatus*, *T. albonubes*, *D. magna*, *n. denticulata sinensis*, *C. pumosus*, *L. hoffmeisteri*, *R. limnocharis*)		Mortality(96 h-LC50); Fry Growth rate	[84]
**Copper sulfate** (for MTT assay 0, 100, 200, 300, 400, 500, 600, 700, 800 μmol/L; for oxdative stress, 0, 100, 200, 300, 400 μmol/L; for Comet assay, 0, 100, 200,400, 800 μmol/L; for 24 h exposure)	Laboratory test; cellular test	Cellular malformation (e.g., indistinct nuclear boundaries loose ribosomes in the cytoplasm); oxidative stress (e.g., SOD activity) Cellular viability		[33]
**Waste including PBDE, metal, and so on**	Field study using caged fish; target organ of liver	Histopathological change (e.g., swelled shape); PBDE accumulation,	Survival rate;	[83]
**17α-ethinylestradiol (EE2), 17β-estradiol (E2)** (0, 1, 10, 100, 1000 ng/L)	Laboratory test	Endocrine disruption		[90]

**Table 6:** Summary of selected promising ecotoxicity test species.

Species	Distribution (Indigenous Region)	Habitat	Ecological Traits	Traits/Suitability to Laboratory Test	Others
*Hydrilla verticillata*	Africa, south Asia, Southeast Asia including Egypt, China, Korea, Japan	temperate and tropical regions such as are spring, leks, marshes, ditches, rivers, tidal zones channels, quarries, shallow reservoirs, and ditches	producer in a tropic level/abundant individual/role as a habitat to small organisms	Submerged plant/well-studied life cycle/phytoremediation capacity absorbing metals/habitat provider to small organism/long growth cycle/large contact surface/tolerant to temp., pH, salinity	Suitable to absorbing/precipitating chemicals
*Scenedesmus obliquus*	world-wide distribution including Egypt, China, Korea, Japan	Wastewater/nutrient-rich water of freshwater or brackishwater	Producer in a tropic level/abundant individual	Rapid growth/easy of culture/capacity of removing metal/tolerant to harsh environment/reproduction with sexual and asexual pattern/well-studied life cycle/euryhalinity	Suitable to environmental monitoring in wastewater and diverse stressful environment /Whole genome sequence
*Neocaridina denticulata*	Korea, China, Taiwan, Vietnam, Japan	Freshwater/lentic and lotic waters	1st consumer in a tropic level/abundant individual	Diverse color/small size (total length < 3 cm)/easy to manipulate/short life cycle/well-studied life cycle/transparent body suitable to observing phenotypes/resistant to bacterial infection pH, temperature, oxygen contents, ammonia, nitrate)/Omnivores	Suitable to genetic study using lots of individuals under a laboratory condition
*Zacco platypus*	Korea, Japan, China, Northern Vietnam	Freshwater/Subtropical region, stream and river having a rapid flow	Benthopelagic species/2st consumer in a tropic level/abundant individual	well-studied life cycle/sexual traits (e.g., nuptial organ, brilliant body color)/Omnivores	Balanced research status between lab. and field/
*Misgurnus anguillicaudatus*	Korea, Japan, China, India, Thailand, Laos, Tugur and amur drainages, Vietnam, Taiwan, Cambodia	Freshwater/subtropical region/Ditches, rice paddy fields, streams, mud places, ponds	Benthic species/2st consumer in a tropic level/abundant individual	Persistent to drought, ammonia/diverse polyploidy population/well-studied life cycle/Omnivores	Suitable to absorbing/precipitating chemicals/Whole genome sequence

**Table 7:** Information for several model ecotoxicity test species listed in international test guideline.

Classification	Species	Genome Information	Guideline	Reference
Vertebrate (Fish)	*Danio rerio*	GRCz11(reference genome) and 17 other assemblies	OECD	[125,139]
Vertebrate (Fish)	*Oncorhynchus mykiss*	USDA_OmykA_1.1(reference genome) and 5 other assemblies	OECD, EPA	[7,125,139]
Vertebrate (Fish)	*Cyprinus carpio*	ASM1834038v1 (reference genome) and 7 other assemblies	OECD	[125,139]
Vertebrate (Fish)	*Oryzias latipes*	ASM223467v1(reference genome) and 31 other assemblies	OECD	[125,139]
Vertebrate (Fish)	*Pimephales promelas*	EPA_FHM_2.0 and 2 other assemblies	OECD, EPA	[7,125,139]
Vertebrate (Fish)	*Salvelinus fontinalis*	ASM2944872v1 (reference genome)	EPA	[7,125]
Vertebrate (Fish)	*Cyprinodon variegatus*	C_variegatus-1.0 (reference genome)	EPA	[7,125]
Vertebrate (Fish)	*Mendia beryllina*	ASM1336337v1 (reference genome)	EPA	[7,125]
Vertebrate (Fish)	*Menidia menidia*	Menidia_menidia_GA_1.0 (reference genome) and 2 other assemblies	EPA	[7,125]
Vertebrate (Fish)	*Menidia Peninsulae*	-	EPA	[7,125]
Vertebrate(Amphibians)	*Xenopus laevis*	Xenopus_laevis_v10.1 (reference genome) and 1 other assembly	OECD	[125,139]
Invertebrate	*Ceriodaphnia dubia*	CSIRO_AGI_Cdub_v0.2 (reference genome)	EPA	[7,125]
Invertebrate	*Mysidopsis bahia*	-	EPA	[7,125]
Invertebrate	*Holmesimysis costata*	-	EPA	[7,125]
Invertebrate	*Daphnia magna*	ASM2063170v1.1 (reference genome) and 5 other assemblies	OECD, EPA	[7,125,139]
Invertebrate	*Daphnia pulex*	ASM2113471v1 (reference genome) and 6 other assemblies	OECD, EPA	[7,125,139]
Invertebrate	*Chironomus*, *riparius*	PGI_CHIRRI_v4 (reference genome) and 7 other assemblies	OECD	[125,139]
Invertebrate	*Chironomus*, *tentans*	idChiTent1.1 (reference genome) and 7 other assemblies	OECD	[125,139]
Invertebrate	*Artemia salina*	-	EPA	[7,125]
algae	*Pseudokirchneriella subcapitata*	-	OECD	[125,139]
algae	*Desmodesmus subspicatus*	-	OECD	[125,139]
*Diatoms*	Navicula pelliculosa	Fpelliculosa_ONT_v02(reference genome) and 1 other assembly	OECD	[125,139]
*Cyanobacteria*	Anabaena flos-aquae	ASM1251639v1	OECD	[125,139]
*Cyanobacteria*	*Synechococcus leopoliensis*	-	OECD	[125,139]
Plant	genus *Lemna(Lemna minuta)*	Salk_lm5633_a03 (reference genome)	OECD	[125,139]

## Data Availability

The data used in this work is included in the article.

## References

[1] Van Leeuwen C.J., Germens J.L.M. (1995). Risk Assessment of Chemicals: An Introduction.

[2] Lee J.W., Won E.J., Raisuddin S., Lee J.S. (2015). Significance of adverse outcome pathways in biomarker-based environmental risk assessment in aquatic organisms. J. Environ. Sci..

[3] ASTM (2019). Standard Guide for Selection of Resident Species as Test Organisms for Aquatic and Sediment Toxicity Tests.

[4] Van den Berg S.J.P., Baveco H., Butler E., Laender D., Focks A., Franco A., Rendal C., Van den Brink P.J. (2019). Modeling the sensitivity of aquatic macroinvertebrates to chemicals using traits. Environ. Sci. Technol..

[5] Cefic The European Chemical Industry Facts and Figures, a Vital Part of Europe’s Future. https://cefic.org/a-pillar-of-the-european-economy/.

[6] Kim J.-S. (2021). A Study on Preparation of Domestic Institutional Improvement Plan through Comparison and Analysis of Domestic and Foreign Chemical Management Systems. Ph.D. Thesis.

[7] US EPA Methods for Measuring the Acute Toxicity of Effluents and Receiving Waters to Freshwater and Marine Organisms, EPA-821-R-02-012. https://www.epa.gov/.

[8] European Commission (2003). echnical Guidance Document on Risk Assessment in Support of Commission Directive 93/67/EEC on Risk Assessment for New Notified Substances and Commission Regulation (EC) No 1488/94 on Risk Assessment for Existing Substances and Directive 98/8/EC of the European Parliament and of the Council Concerning the Placing of Biocidal Products on the Market.

[9] Ishaq A.G., Matias-Peralta H.M., Basri H. (2016). Bioactive compounds from green microalga-*Scenedesmus* and its potential applications: A brief review. Pertanika J. Trop. Agric. Sci..

[10] Zhang Y., Ren L., Chu H., Zhou X., Yao T., Zhang Y. (2019). Optimization for *Scenedesmus obliquus* cultivation: The effects of temperature, light intensity and pH on growth and biochemical composition. Microbiol. Biotechnol. Lett..

[11] Zhang Y., Meng T., Shi L., Guo X., Si X., Yang R., Quan X. (2019). The effects of humic acid on the toxicity of graphene oxide to *Scenedesmus obliquus* and *Daphnia magna*. Sci. Total Environ..

[12] Ji M.-K., Yun H.-S., Park Y.-T., Kabra A.N., Oh I.-H., Coi J. (2015). Mixotrophic cultivation of a microalga *Scenedesmus obliquus* in municipal wastewater supplemented with food wastewater and flue gas CO_2_ for biomass production. J. Environ. Manag..

[13] Gris B., Morosinotto T., Giacometti G.M., Bertucco A., Sforza E. (2014). Cultivation of *Scenedesmus obliquus* in photobioreactors: Effects of light intensities and light-dark cycles on growth, productivity, and biochemical composition. Appl. Biochem. Biotechnol..

[14] Trainor F.R. (1996). Reproduction in *Scenedesmus*. Algae.

[15] Patrick A.E.S., Florentine S. (2021). Factors affecting the global distribution of *Hydrilla verticillata* (L. fil.) Royle: A review. Weed Res..

[16] Gao J., Liu C., Zhang J., Zhu S., Shen Y., Zhang R. (2018). Effect of fluoride on photosynthetic pigment content and antioxidant system of *Hydrilla verticillata*. Int. J. Phytoremediat..

[17] Rojas-Sandoval J. (2016). Hydrilla verticillata (hydrilla). Invasive Species Compendium.

[18] Madeira P.T., Van T.K., Steward K.K., Schnell R.J. (1997). Random amplified polymorphic DNA analysis of the phenetic relationships among world-wide accessions of *Hydrilla verticillata*. Aquat. Bot..

[19] Okupnik A., Pflugmacher S. (2016). Oxidative stress response of the aquatic macrophyte *Hydrilla verticillata* exposed to TiO_2_ nanoparticles. Environ. Toxicol. Chem..

[20] Ceschin S., Bellini A., Scalici M. (2021). Aquatic plants and ecotoxicological assessment in freshwater ecosystems: A review. Environ. Sci. Pollut. Res..

[21] Nur F.A.H., Christianus A. (2013). Breeding and life cycle of *Neocaridina denticulata* sinensis (Kemp, 1918). Asian J. Anim. Vet. Adv..

[22] Oh C.W., Ma C.W., Hartnoll R.G., Suh H.L. (2003). Reproduction and population dynamics of the temperate freshwater shrimp, *Neocaridina denticulata denticulata* (De Haan, 1844), in a Korean stream. Crustaceana.

[23] Mahmoud H.H.A., Sastranegara M.H., Kusmintarsh E.S. (2020). The lifecycle of *Neocaridina denticulata* and *N. palmata* in aquariums. Biodivers. J..

[24] Mykles D.L., Hui J.H.L. (2015). *Neocaridina denticulata*: A decapod crustacean model for functional genomics. Integr. comp. Biol..

[25] Levitt-Barmats Y., Yanani Z., Cohen T.M., Shenkar N. (2019). Life-history traits and ecological characteristics of the ornamental shrimp *Neocaridina denticulta* (De Haan, 1844), recently introduced into the freshwater systems of Israel. Aquat. Invasions.

[26] Onuki K., Fuke Y. (2022). Rediscovery of a native freshwater shrimp, *Neocaridina denticulata*, and expansion of an invasive species in lake Biwa, Japan: Genetic and morphological approach. Research Square 1-22. Conserv. Genet..

[27] Sung H.-H., Lin Y.-H., Hsiao C.-Y. (2011). Differential immune responses of the green neon shrimp (*Neocaridina denticulata*) to dipropyl phthalate. Fish Shellfish Immunol..

[28] Froese R., Pauly D. Fishbase. World Wide Web Electronic Publication. http://www.fishbase.org/.

[29] Kim J.H., Yeom D.H. (2009). Population response of Pale Chub (*Zacco platypus*) exposed to wastewater effluents in Gap stream. Toxicol. Environ. Health Sci..

[30] Kim H.C., Kim M., Yu H. (1994). Biological control of vector mosquitoes by the use of fish predators, *Moroco oxycephalus* and *Misgurnus anguillicaudatus* in the laboratory and semi-field rice paddy. Korean J. Entomol..

[31] Lei F., Wang B. (1990). Studies on the reproduction and growth of loach. Acta Hydrobiol. Sin..

[32] Suzuki R. (1983). Multiple spawning of the cyprinid loach, *Misgurnus anguillicaudatus*. Aquaculture.

[33] Qin T., Li X., Yang Y., Li Z., Liang Y., Zhang X., Jiang S. (2018). Toxic effects of copper sulfate on diploid and triploid fin cell lines in *Misgurnus anguillicaudatus*. Sci. Total Environ..

[34] Zhang Y., Sun Q., Zhou J., Masunaga S. (2015). Reduction in toxicity of wastewater from three wastewater treatment plants to alga (*Scenedesmus obliquus*) in northeast China. Ecotox. Environ. Saf..

[35] Cheng C., Huang L., Ma R., Zhou Z., Diao J. (2015). Enantioselective toxicity of lactofen and its metabolites in *Scenedesmus obliquus*. Algal Res..

[36] Li Z., Juneau P., Lian Y., Zhang W., Wang S., Wang C., Shu L., Yan Q., He Z., Xu K. (2020). Effects of titanium dioxide nanoparticles on photosynthetic and antioxidative processes of *Scenedesmus obliquus*. Plants.

[37] Liu Y., Wang Z., Wang S., Fang H., Ye N., Wang D. (2019). Ecotoxicological effects on *Scenedesmus obliquus* and *Danio rerio* co-exposed to polystyrene nano-plastic particles and natural acidic organic polymer. Environ. Toxicol. Pharm..

[38] Qing C., Zhang H., Chen A., Lin Y., Shao J. (2020). Effects and possible mechanisms of sanguinarine on the competition between *Raphidiopsis raciborskii* (Cyanophyta) and *Scenedesmus obliquus* (Chlorophyta): A comparative toxicological study. Ecotox. Environ. Saf..

[39] Li Z., Chen J., Chen J., Jin J., Chen H., Liu H. (2021). Metabolomic analysis of *Scenedesmus obliquus* reveals new insights into the phytotoxicity of imidazolium nitrate ionic liquids. Sci. Total Environ..

[40] Zhang L., Goswami N., Xie J., Zhang B., He Y. (2017). Unraveling the molecular mechanism of photosynthetic toxicity of highly fluorescent silver nanoclusters to *Scenedesmus obliquus*. Sci. Rep..

[41] Xia Y., Liu D., Dong Y., Chen J., Liu H. (2018). Effect of ionic liquids with different cations and anions on photosystem and cell structure of *Scenedesmus obliquus*. Chemosphere.

[42] Wang H., Jin M., Mao W., Chen C., Fu L., Li Z., Du S., Liu H. (2020). Photosynthetic toxicity of non-steroidal anti-inflammatory drugs (NSAIDs) on green algae *Scenedesmus obliquus*. Sci. Total Environ..

[43] Bhuvaneshwari M., Iswary V., Archanaa S., Madhu G.M., Suraish Kumar G.K., Nagarajan R., Chandrasekaran N., Mukherjee A. (2015). Cytotoxicity of ZnO Nos towards fesh water algae Scenedemus obliquus at low exposure concentrations in UV-C, visible and dark conditions. Aquat. Toxicol..

[44] Liu W., Zhang Y.-B., Quan X., Jin Y.-H., Chen S. (2009). Effect of perfluorooctane sulfonate on toxicity and cell uptake of other compounds with different hydrophobicity in green alga. Chemosphere.

[45] Wang P., Zhang B., Zhang H., He Y., Ong C.N., Yang J. (2019). Metabolites change of Scenedesmus obliquus exerted by AgNPs. J. Environ. Sci..

[46] Ma S., Li D., Yu Y., Li D., Yadav R.S., Feng Y. (2019). Application of a microalga, *Scenedesmus obliquus* PF3, for the biological removal of nitric oxide (NO) and carbon dioxide. Environ. Pollut..

[47] Chao W., Guanghua L., Zhuyun T., Xiaoling G. (2008). Quantitative structure-activity relationships for joint toxicity of substituted phenols and anilines to Scenedesmus obliquus. J. Environ. Sci..

[48] Nadaraja A.V., Saraswathy D.P., Ravindran S.C., Mariya A., Russel J.G., Selvanesan P., Pereira B., Bhaskaran K. (2017). Spatio-temporal distribution of percholarte and its toixicity in *Hydrilla verticillata*. Ecotox. Environ. Saf..

[49] Weerakoon H.P.A.T., Atapaththu K.S.S., Asanthi H.B. (2018). Toxicity evaluation and environmental risk assessment of 2-methyl-4-chlorophenoxy acetic acid (MCPA) on non-target aquatic macrophyte *Hydrilla verticillata*. Environ. Sci. Pollut. Res..

[50] Zhang H., Zhang L.-L., Li J., Chen M., An R.-D. (2020). Comparative study on the bioaccumulation of lead, cadmium and nickel and their toxic effects on the growth and enzyme defense strategies of a heavy metal accumulator, *Hydrailla verticillata* (L.F.) Royle. Environ. Sci. Pollut. Res..

[51] Yu G., Huang S., Luo X., Zhao W., Zheng Z. (2023). Single and combined toxicity effects of nanoplastics and bisphenol F on submerged the macrophyte *Hydrilla verticillata*. Sci. Total Environ..

[52] Rozentsvet O.A., Nesterov V.N., Sinyutina N.F. (2012). The effect of copper ions on the lipid composition of subcellular membranes in *Hydrilla verticillata*. Chemosphere.

[53] Yan S., Zhou Q. (2011). Toxic effects of Hydrilla verticillate exposed to toluene, ethylbenzene and xylene and safety assessment for protecting aquatic macrophytes. Chemosphere.

[54] Song Y., He X.J., Chen M., Zhang L.L., Li J., Deng Y. (2018). Effects of pH on the submerged macrophyte *Hydrilla verticillata*. Rus. J. Plant Physiol..

[55] Shi D., Kai Z., Yan X., Changhua Z., Chen C., Zhbing H., Zhenguo S. (2017). *Hydrilla verticillata* employs two different ways to affect DNA methylation under excess copper stress. Aquat. Toxicol..

[56] Jiang H.S., Yin L., Ren N.N., Xian L., Zhao S., Li W., Gontero B. (2017). The effect of chronic silver nanoparticles on aquatic system in microcosms. Environ. Pollut..

[57] Shi D., Zhuang K., Chen Y., Hu Z., Shen Z. (2021). Phytotoxicity and accumulation of Cu in mature and young leaves of submerged macrophyte *Hydrilla verticillata* (L.F.) royle. Ecotox. Environ. Saf..

[58] Li X.-Q., Hua Z.-L., Zhang J.-Y., Gu L. (2022). Ecotoxicological responses and removal of submerged macrophyte *Hydrilla verticillate* to multiple perfluoroalkyl acid (PFAA) pollutants in aquatic environments. Sci. Total Environ..

[59] Huang D.-J., Chen H.-C. (2004). Effects of chlordane and lindane on testosterone and vitellogenin levels in Green neon shrimp (*Neocaridina denticulata*). Int. J. Toxicol..

[60] Sung H.-H., Chiu Y.-W., Wang S.-Y., Chen C.-M., Huang D.-J. (2014). Acute toxicity of mixture of acetaminophen and ibuprofen to green neon shrimp, *Neocaridina denticulata*. Environ. Toxicol. Pharm..

[61] Shin Y.J., Lee J.W., Kim J., Cho J., Kim J.H., Kang M., Kim K., Kim P.J., Park K. (2019). Ring test as acute toxicity test with Korean Freshwater shrimp, *Neocaridina denticulata* using 3,4-Dichloroaniline. J. Environ. Health Sci..

[62] Liu C.-L., Sung H.-H. (2011). Genes are differentially expressed at transcriptional level of *Neocaridina denticulata* following short-term exposure to nonylphenol. Bullet. Environ. Contam. Toxicol..

[63] Hong L., Li M.-H. (2007). Acute toxicity of 4-Nonylphenol to aquatic invertebrates in Taiwan. Bull. Environ. Contam. Toxicol..

[64] Siregar P., Suryanto M.E., Chen K.H.-C., Huang J.-C., Chen H.-M., Kurnia K.A., Santoso F., Hussain A., Hieu B.T.N., Saputra F. (2021). Exploiting the freshwater shrimp *Neocaridina denticulata* as aquatic invertebrate model to evaluate nontargeted pesticide induced toxicity by investigating physiologic and biochemical parameters. Antioxidants.

[65] Li Z., Murray M.-Y. (2019). In vitro micro-tissue and -organ models for toxicity testing. Comprehensive Biotechnology.

[66] Wu J.-P., Li M.-H. (2015). Inhibitory effects of pain relief drugs on neurological enzymes: Implications on their potential neurotoxicity to aquatic animals. Environ. Toxicol. Pharm..

[67] Huang D.-J., Chen H.-C., Wu J.-P., Wang S.-Y. (2006). Reproduction obstacles for the female green neon shrimp (*Neocaridina denticulata*) after exposure to chlordane and lindane. Chemosphere.

[68] Zhang R., Wang Y., Chen F., Yu Q., Sun Y., Zhang J. (2022). Characterization of peroxiredoxin from *Neocaridina denticulata sinensis* and its antioxidant and DNA protection activity analysis. Fish Shellfish Immunol..

[69] Xing K., Liu Y., Yan C., Zhou Y., Sun Y., Su N., Yang F., Xie S., Zhang J. (2021). Transcriptome analysis of *Neocaridina denticulate* sinensis under copper exposure. Gene.

[70] Xing K., Liu Y., Yan C., Zhou Y., Zhang R., Sun Y., Zhang J. (2022). Transcriptomic analysis of *Neocaridina denticulata* sinensis hepatopancreas indicates immune changes after copper exposure. Fish Shellfish Immunol..

[71] Kim J.H., Yeom D.-H., Kim W.-K., An K.-G. (2016). Reginal ecological health or risk assessments of stream ecosystems using biomarkers and bioindicators of target species (*Pale Chub*). Wat. Air Soil Poll..

[72] Kim W.-K., Jung J. (2016). In situ impact assessment of wastewater effluents by integrating multi-level biomarker responses in the plae chub (*Zacco platypus*). Ecotox. Environ. Saf..

[73] Jeon H.-J., Park Y.-C., Lee W.-O., Lee J.-H., Kim J.-H. (2011). cDNA cloning and expression of a cytochrome P4501A(CYP1A) from the Pale chub, *Zacco platypus*. Korean J. Limnol..

[74] Samanta P., Im H., Yoo J., Lee H., Kim N.-Y., Kim W., Hwang S.-J., Kim W.-K., Jung J. (2018). Comparative assessment of the adverse outcome of wastewater effluents by integrating oxidative stress and histopathological alterations in endemic fish. J. Hazard. Mater..

[75] Kawabata Z., Yoshida T., Nakagawa H. (1997). Effect of ammonia on the survival of *Zacco platypus* (Temminck and schlegel) at each developmental stage. Environ. Pollut..

[76] Lee S., Kim C., Kim J., Kim W.K., Shin H.S., Lim E.S., Lee J.W., Kim S., Kim K.T., Lee S.K. (2015). Cloning metallothionein gene in *Zacco platypus* and its potential as an exposure biomarker against cadmium. Environ. Monit. Assess..

[77] Lee J.W., Kim Y.H., Yoon S., Lee S.K. (2014). Cytochrome P450 system expression and DNA adduct formation in the liver of *Zacco platypus* following waterborne Benzo(a)pyrene exposure: Implications for biomarker determination. Environ. Toxicol..

[78] Park C.B., Kim G.-E., Kim D.-W., Kim S., Yeom D.-H. (2021). Biomonitoring the effects of urban-stream waters on the health status of pale chub (*Zacco platypus*): A comparative analysis of biological indexes and biomarker levels. Ecotox. Environ. Saf..

[79] Lee J.W., Yoon H.G., Lee S.K. (2015). Benzo(a)pyrene-induced cytochrome p4501A expression of four freshwater fishes (*Oryzias latipes*, *Danio rerio*, *Cyprinus carpio*, and *Zacco platypus*). Environ. Toxicol. Pharm..

[80] Kim W.-S., Park K., Park J.-W., Lee S.-H., Kim J.-H., Kim Y.-J., Oh G.-H., Ko B.-S., Park J.-W., Hong C. (2022). Transcriptional responses of stress-related genes in pale chub (*Zacco platypus*) inhabiting different aquatic environments: Application for biomonitoring aquatic ecosystems. Int. J. Environ. Res. Public Health.

[81] Hayashi M., Ueda T., Uyeno K., Wada K., Kinae N., Saotome K., Tanaka N., Takai A., Sasaki Y.F., Asano N. (1998). Development of genotoxicity assay systems that use aquatic organisms. Mutat. Res..

[82] Gao J., Wang F., Jiang W., Miao J., Wang P., Zhou Z., Liu D. (2020). A full evaluation of chiral phenylpyrazole pesticide flufiprole and the metabolites to non-target organism in paddy field. Environ. Pollut..

[83] Xiaofei Q., Xijuan X., Yan L., Yaxian Z., Zhongzhi Y., Shan F., Mi T., Xingru Z., Zhanfen Q., Xiaobai X. (2009). Ecotoxicological effects of mixed pollutants resulted from e-wastes recycling and bioaccumulation of polybrominated diphenyl ethers in Chinese loach (*Misgurnus anguillicaudatus*). J. Environ. Sci..

[84] Wang X.-N., Liu Z.-T., Yan Z.-G., Zhang C., Wang W.-L., Zhou J.-L., Pei S.-W. (2013). Development of aquatic life criteria for triclosan and comparison of the sensitivity between native and non-native species. J. Hazard. Mater..

[85] Xia X., Xia X., Huo W., Dong H., Zhang L., Chang Z. (2016). Toxic effects of imidacloprid on adult loach (*Misgurnus anguillicaudatus*). Environ. Toxicol. Pharmcol..

[86] Nan P., Yan S., Li L., Chen J., Du Q., Chang Z. (2015). Toxicity effect of dichlorvos on loach (*Misgurnus anguillicaudatus*) assessed by micronucleus test, hepatase activity analysis and comet assay. Toxicol. Ind. Health.

[87] Huo W., Wan R., Wang P., Zhang L., Xia X. (2019). Molecular cloning, characterization of dax1 gene and its response to progesterone in *Misgurnus anguillicaudatus*. Drug Chem. Toxicol..

[88] Qin Y., Li X., Xiang Y., Wu D., Bai L., Li Z., Liang Y. (2017). Toxic effects of glyphosate on diploid and triploid fin cell lines from *Misgurnus anguillicaudatus*. Chemosphere.

[89] Wu H., An P., Wang J., Guan R., Yang N., Lei X. (2021). Effects of phenanthrene stress on gonads and vitellogenin of Loach (*Misgurnus anguillicaudatus*). Bull. Environ. Contam. Toxicol..

[90] Wu M., Nishimiya O., Nakamori M., Soyano K., Todo T., Hara A., Hiramatsu N. (2014). Molecular cloning and characterization of the expression profiles of vitellogenin transcripts in the Dojo Loach (*Misgurnus anguillicaudatus*) in response to 17α-estradiol administration. Zool. Sci..

[91] Li H., Kang M., Sun S., Gao J., Jia Z., Cao X. (2022). Cloning and expressions of chop in loach (*Misgurnus anguillicaudatus*) and its response to hydrogen peroxide (H_2_O_2_) stress. Fish Physiol. Biochem..

[92] Ajala S.O., Alexander M.L. (2020). Assessment of *Chlorella vulgaris*, *Scenedesmus obliquus*, and *Oocystis minuta* for removal of sulfate, nitrate, and phosphate in wastewater. Int. J. Energy Environ. Eng..

[93] Pachés M., Martinez-Guijarro R., Gonzalez-Camejo J., Seco A., Barat R. (2020). Selecting the most suitable microalgae species to treat the effluent from an anaerobic membrane bioreactor. Environ. Technol..

[94] Jia X., Pan Y., Zhu X. (2023). Salinization and heavy metal cadmium impair growth but have contrasting effects on defensive colony formation of *Scenedesmus obliquus*. Sci. Total Environ..

[95] Huang C.-W., Chu P.-Y., Wu Y.-F., Chan W.-P., Wang Y.-H. (2020). Identification of functional SSR markers in freshwater ornamental shrimps *Neocaridina denticulata* using transcriptome sequencing. Mar. Biotechnol..

[96] Sin Y.W., Kenny N.J., Qu Z., Chan K.W., Chan K.W.S., Cheong S.P.S., Leung R.W.T., Chan T.F., Bendena W.G., Chu K.H. (2015). Identification of putative ecdysteroid and juvenile hormone pathway genes in the shrimp *Neocaridina denticulata*. Gen. Comp. Endocrinol..

[97] Liang M., Ma L., Li X., Feng D., Zhang J., Sun Y. (2022). Identification and characterization of two types of triacylglycerol lipase genes from *Neocaridina denticulata sinensis*. Fish Shellfish Immunol..

[98] Baek H.-M., Song H.-B., Cho D.-H. (2006). Reproductive ecology of the Pale chub, *Zacco platypus* in a tributary to the Han river. Korean J. Ichtyol..

[99] Fu C., Fu S.-J., Yuan X.-Z., Cao Z.-D. (2015). Predator-driven intra-species variation in locomotion, metabolism and water velocity preference in pale chub (*Zacco playptus*) along a river. J. Exp. Biol..

[100] Hussein M.N.A., Cao X., Elokil A.A., Huang S. (2020). Characterization of stem and proliferating cells on the retina and lens of loach *Misgurnus anguillicaudatus*. J. Fish Biol..

[101] Tsui T.K.N., Randoall D.J., Hanson L., Farrell A.P., Chew S.F., Ip Y.K. (2004). Dogmas and controversies in the handling of nitrogenous wastes: Ammonia tolerance in the oriental watherloach *Misgurnus anguillicaudatus*. J. Exp. Biol..

[102] ECHA (2020). The Use of Alternatives to Testing on Animals for the REACH Regulation, Fourth Report Under Article 117(3) of the Reach Regulation.

[103] Madden J.C., Enoch S.J., Paini A., Cronin M.T.D. (2020). A review of in Silico tools as alternatives to Animal testing: Principles, resources and applications. Altern. Lab. Anim..

[104] Rehberger K., Kropf C., Segner H. (2018). In vitro or not in vitro: A short journey through a long history. Environ. Sci. Eur..

[105] UK Annual National Statistics Report, Annual Statistics of Scientific Procedures on Living Animals Great Britain 2021 Presented to Parliament Pursuant to Section 21(7) and 21A(1) of the Aniamls (Scientific Procedures) act 1986; Ordered by the House of Commons to be Printed 30, June 2022. http://www.understandinganimal;org.uk/files/9514/9993/4810/annual-statistics-scientific-procedures-living-animals-2016.pdf/.

[106] Lee J.W., Park S., Jang S.-W., Lee S., Moon S., Kim H., Kim P., Yu S.D., Seong C.H. (2019). Toxicity prediction using three quantitative structure-activity relationship (QSAR) programs (TOPKAT^®^, Derek^®^, OECD toolbox). J. Environ. Health Sci..

[107] Chatterjee M., Roy K. (2021). Prediction of aquatic toxicity of chemical mixtures by the QSAR approach using 2D structural descriptors. J. Hazard. Mater..

[108] Lunghini F., Marcou G., Azam P., Enrici M.H., Van Miert E., Varnek A. (2020). Consensus QSAR models estimating acute toxicity to aquatic organisms from different trophic levels: Algae, Daphnia and fish. SAR QSAR Environ. Res..

[109] Krishna G., Krishna P.A., Goel S., Krishna K.A., Gupta R.C. (2019). Alternative animal toxicity testing and biomarkers. Biomarkers in Toxicology.

[110] Rosner A., Armengaud J., Ballarin L., Barnay-Verdier S., Cima F., Coelho A.V., Domart-Coulon I., Drobne D., Geneviere A.-M., Kokalj A.J. (2021). Stem cells of aquatic invertebrates as an advanced tool for assessing ecotoxicological impacts. Sci. Total Environ..

[111] Hamm J., Sullivan K., Clippinger A.J., Strickland J., Bell S., Bhhatarai B., Blaauboer B., Casey W., Dorman D., Forsby A. (2017). Alternative approaches for identifying acute systemic toxicity: Moving from research to regulatory testing. Toxicol. In Vitro.

[112] Teixido E., Leuthold D., de Croze N., Leonard M., Scholz S. (2020). Comparative assessment of the sensitivity of fish early-life stage, Daphnia, and algae tests to the chronic ecotoxicity of xenobiotics: Perspectives for alternatives to animal testing. Environ. Toxicol. Chem..

[113] Sobanska M., Scholz S., Nyman A.M., Cesnaitis R., Gutierrez Alonso S., Kluver N., Kuhne R., Tyle H., de Knecht J., Dang Z. (2018). Applicability of the fish embryo acute toxicity (FET) test (OECD 236) in the regulatory context of registration, evaluation, authorization, and restriction of chemicals (REACH). Environ. Toxicol. Chem..

[114] Krzykwa J.C., King S.M., Jeffries M.K.S. (2021). Investigating the predictive power of three potential sublethal endpoints for the Fathead minnow fish embryo toxicity test: Snout-Vent length, eye size, and Pericardial edema. Environ. Sci. Technol..

[115] Schirling M., Bohlen A., Triebskorn R., KÖhler H.-R. (2006). An invertebrate embryo test with the apple snail *Marisa cornuarietis* to assess effects of potential developmental and endocrine disruptors. Chemosphere.

[116] Liu C., Du T., Zhou B. (2007). Evaluation of estrogenic activities and mechanism of action of perfluorinated chemicals determined by vitellogenin induction in primary cultured tilapia hepatocytes. Aquat. Toxicol..

[117] Liu C., Yu K., Shi X., Wang J., Lam P.K.S., Wu R.S.S., Zhou B. (2007). Induction of oxidative stress and apoptosis by PFOS and PFOA in primary cultured hepatocytes of freshwater tilapia (*Oreocromis niloticus*). Aquat. Toxicol..

[118] Rymuszka A., Sieroslawska A., Adaszek Ł. (2021). Cytotoxic and immunological responses of fish leukocytes to nodularin exposure in vitro. J. Appl. Toxicol..

[119] Ladhar-Chaabouni R., Ayadi W., Sahli E., Mokdad-Gargouri R. (2021). Establishment of primary cell culture of *Ruditapes decussatus* haemocytes for metal toxicity assessment. In Vitro Cell. Dev. Biol. Anim..

[120] Tanneberger K., Knöbel M., Busser F.J.M., Sinnige T.L., Hermens J.L.M., Schirmer K. (2013). Predicting fish acute toxicity using a fish gill cell line-based toxicity assay. Environ. Sci. Technol..

[121] Lillicrap A., Belanger S., Burden N., Du Pasquier D., Embry M.R., Halder M., Lampi M.A., Lee L., Norberg-king T., Rattner B.A. (2016). Alternative approaches to vertebrate ecotoxicity tests in the 21st century: A review of developments over the last 2 decades and current status. Environ. Toxicol. Chem..

[122] Hasin Y., Seldin M., Lusis A. (2017). Multi-omics approaches to disease. Genome Biol..

[123] Likic V.A., McConville M.J., Lithgow T., Bacic A. (2010). Systems biology: The next frontier for bioinformatics. Adv. Bioinform..

[124] Lee H., Sung E.J., Seo S., Min E.K., Lee J.-Y., Shim I., Kim P., Kim T.-Y., Lee S., Kim K.-T. (2021). Integrated multi-omics analysis reveals the underlying molecular mechanism for developmental neurotoxicity of perfluorooctanesulfonic acid in zebrafish. Environ. Int..

[125] NCBI National Library of Medicine, Genome. https://www.ncbi.nlm.nih.gov/datasets/genome/.

[126] Loerracher A.-K., Braunbeck T. (2021). Cytochrome P450 dependent biotransformation capacities in embryonic, juvenile and adult stages of zebrafish (Danio rerio)-a state of the art review. Arch. Toxicol..

[127] Rojas-Hernandez N., Veliz D., Vega-Retter C. (2019). Selection of suitable reference genes for gene expression analysis in gills and liver of fish under field pollution conditions. Sci. Rep..

[128] Jin Y., Liu F., Huang W., Sun Q., Huang X. (2019). Identification of reliable reference genes for qRT-PCR in the ephemeral plant Arabidopsis pumila based on full-length transcriptome data. Sci. Rep..

[129] Heckmann L.-H., Connon R., Hutchinson T.H., Maund S.J., Sibly R.M., Callaghan A. (2006). Expression of target and reference genes in Daphnia magna exposed to ibupropen. BMC Genom..

[130] European Commission (2021). Regulation (EC) No 1907/2006 of the European Parliamentand of the Council of 18 December 2006 Concerning the Registration Evaluation, Authorization and Restriction of Chemicals (REACH), Establishing a European Chemicals Agency, Amending Directive 1999/45/eC and Repealing Council Regulation (EEC) No 793/93 and Commission Regulation (EC) No 1488/94 as Well as Council Directive 76/769/”EEC and Commission Directives 91/155/EEC, 93/67/EEC, 93/67/EEC, 93/105/EC and 200/21/EC.

[131] Lee J.W., Won E.-J., Kang H.-M., Hwang D.-S., Kim D.-H., Kim R.-K., Lee S.-J., Lee J.-S. (2016). Effects of multi-walled carbon nanotube (MWCNT) on antioxidant depletion, the ERK signaling pathway, and copper bioavailability in the copepod (*Tigriopus japonicus*). Aquat. Toxicol..

[132] Lee J.W., Lee J.-W., Kim K., Shin Y.J., Kim J., Kim S., Kim H., Kim P., Park K. (2017). PFOA-induced metabolism disturbance and multi-generational reproductive toxicity in Oryzias latipes. J. Hazard. Mater..

[133] Kavlock R.J., Bahadori T., Barton-Maclaren T., Gwinn M.R., Rasenberg M., Thomas R.S. (2018). Accelerating the pace of chemical risk assessment. Chem. Res. Toxicol..

[134] OECD (2020). Overview of Concepts and Available Guidance Related to Integrated Approaches to Testing and Assessment (IATA).

[135] Kim B., Moran N.P., Reinhold K., Sánchez-Tójar A. (2021). Male size and reproductive performance in three species of livebearing fishes (*Gambusia* spp.): A systematic review and meta-analysis. J. Anim. Ecol..

[136] Freitas E.C., Rocha O. (2021). Acute toxicity tests with the tropical cladocerans *Pseudosida ramosa*: The importance of using native species as test organisms. Arch. Environ. Contam. Toxicol..

[137] Wang P., Ng Q.X., Zhang H., Zhang B., Ong C.N., He Y. (2018). Metabolite changes behind faster growth and less reproduction of *Daphnia similis* exposed to low-dose silver nanoparticles. Ecotox. Environ. Saf..

[138] Zhong G., Wu Z., Yin J., Chai L. (2018). Responses of *Hydrilla verticillata* (L.F.) royle and *Vallisneria natans* (Lour.) Hara to glyphosate exposure. Chemosphere.

[139] OECD Guidelines for the Testing of Chemicals, Section 2, Effects on Biotic Systems. https://www.oecd-ilibrary.org/environment/oecd-guidelines-for-the-testing-of-chemicals-section-2-effects-on-biotic-systems_20745761.

[140] Lee J.W., Lee J.-W., Kim K., Shin Y.-J., Kim J., Kim H., Kim H., Min S.-A., Kim P., Choi J. (2019). n-Butyl acrylate-induced antioxidant system alteration through two generations in *Oryzias latipes*. Fish Physiol. Biochem..

[141] Lee J.W., Choi K., Park K., Seong C., Yu S.D., Kim P. (2020). Adverse effects of perfluoroalkyl acids on fish and other aquatic organisms: A review. Sci. Total Environ..

[142] Ritschar S., Rabus M., Laforsch C. (2019). Predator-specific inducible morphological defenses of a water flea against two freshwater predators. J. Morphol..

[143] Rodgher S., Espindola E.L.G., Lombardi A.T. (2010). Suitability of *Daphnia similis* as an alternative organism in ecotoxicological tests: Implications for metal toxicity. Ecotoxicology.

